# Cu/ZnO@GO promoted green synthesis of novel dipyridopyrimidines: evaluation of biological activity and theoretical study of the mechanism using a DFT method

**DOI:** 10.1039/d5ra04054j

**Published:** 2025-08-26

**Authors:** Elham Ezzatzadeh, Nasrin Karami Hezarcheshmeh, Reza Akbari

**Affiliations:** a Department of Chemistry, Ard.C., Islamic Azad University Ardabil Iran Elham.Ezzatzadeh@iau.ac.ir dr.ezzatzadeh@yahoo.com; b Department of Chemistry, SR.C., Islamic Azad University Tehran Iran; c Department of Chemistry, Faculty of Basic Sciences, Gonbad Kavous University Gonbad Kavous Iran

## Abstract

This investigation presents a single-step reaction performed at ambient temperature in aqueous media, involving acetylacetone, aldehydes, guanidine, and activated acetylenic compounds, employing a catalytic system consisting of small quantities of Cu/ZnO@GO. Currently, the antioxidant potential of select synthesized dipyridopyrimidines is evaluated *via* diphenyl-picrylhydrazine (DPPH) radical scavenging assays. Furthermore, the antimicrobial efficacy of the synthesized compounds was systematically assessed using the disk diffusion method, which involved testing against two distinct strains of Gram-negative bacteria and Gram-positive bacteria. In a separate vein, the catalytic efficacy of the Cu/ZnO@GO catalyst was rigorously assessed in the reduction of organic pollutants, specifically 4-nitrophenol (4-NP), in aqueous solutions under benign conditions. The data revealed that nanocomposites prepared *via* a biosynthetic method demonstrated remarkable catalytic performance in the remediation of organic contaminants, achieving substantial reduction within mere seconds. The synthetic methodology employed for the generation of dipyridopyrimidines was characterized by a confluence of advantageous attributes, encompassing accelerated reaction kinetics, elevated product yields, and facile recovery of the catalyst from the reaction milieu. Density Functional Theory (DFT) calculations at the B3LYP/6-311G(d,p) level were conducted to explore the reaction mechanism, employing the total energy of reactants and products as a basis for its determination.

## Abstract 2

This investigation presents a multicomponent reaction involving acetylacetone, aldehydes, guanidine, and activated acetylenic compounds in water at room temperature employing small quantities of Cu/ZnO@GO as nanocatalyst.

## Graphical abstract

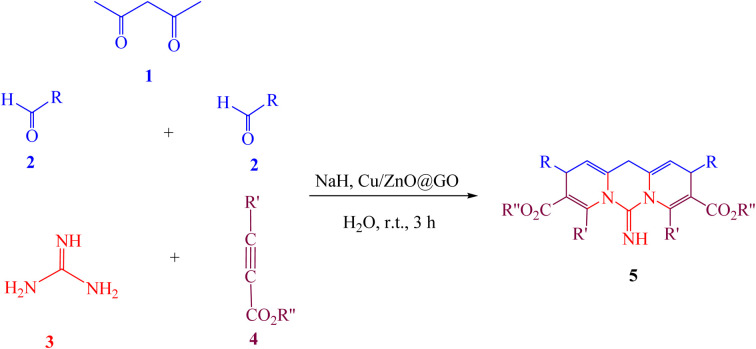

## 1. Introduction

**Scheme 1:**
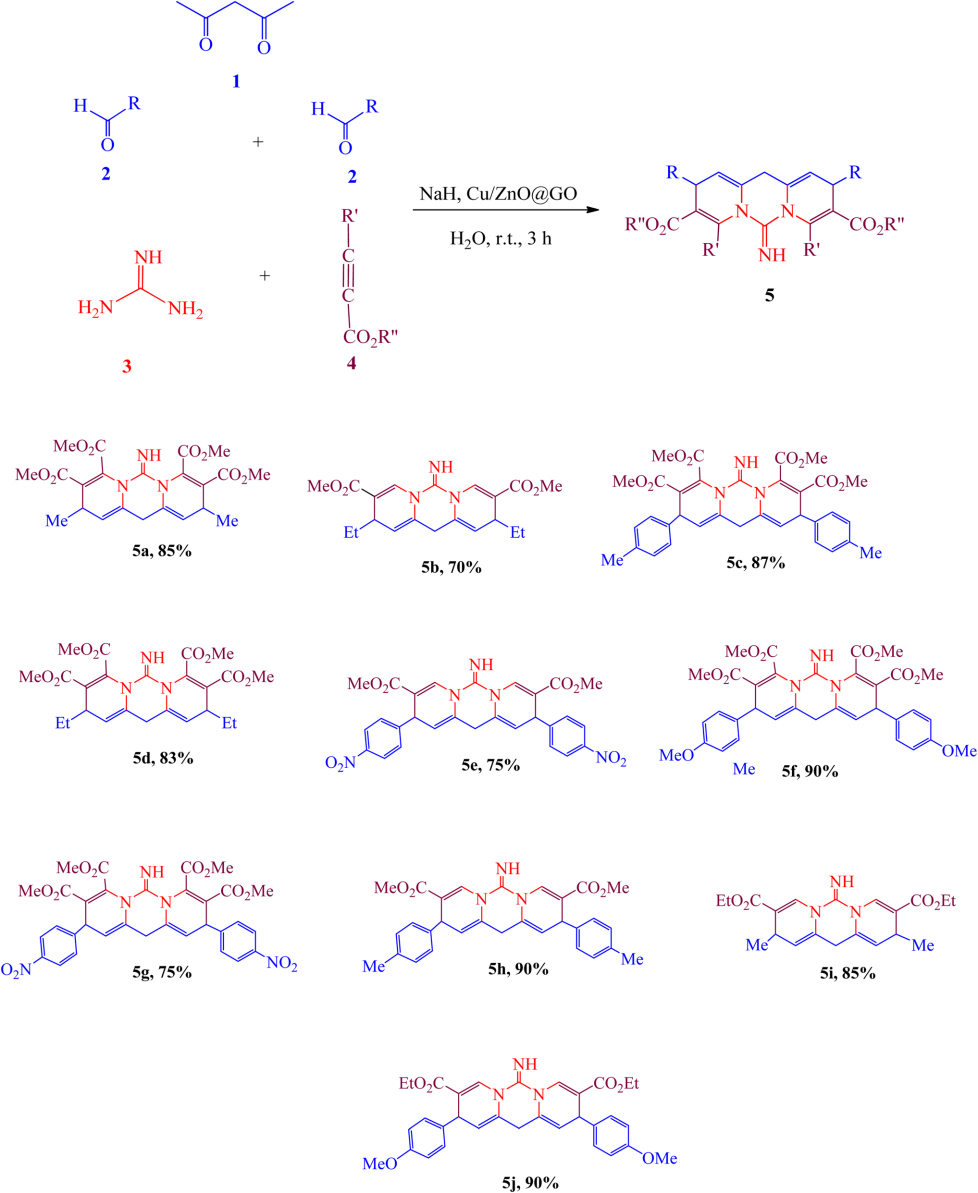
Synthesis of dipyridopyrimidines 5.

Multicomponent reactions (MCRs) are a prevalent methodology for the synthesis of biologically active compounds.^1–5^ In the context of heterocyclic compound production, MCRs demonstrate superior efficacy compared to alternative methodologies, esteemed as they are for their efficient yields and atom economy.^6–8^ These heterocycles, readily sourced from natural origins and fundamentally important to biological processes, are found as structural components in a plethora of naturally occurring materials, including vitamins, hormones, and antibiotics. Consequently, the development of effective strategies for their synthesis is an area of considerable interest within the field of synthetic organic chemistry.^9^ Nitrogen-containing heterocyclic compounds, particularly, are of considerable value in medicinal chemistry, exerting a beneficial influence across numerous facets of daily life. Given their diverse biological functionalities and significant roles in medicinal applications, heterocyclic compounds are recognized as exceptionally important organic molecules,^10–21^ resulting in the establishment of myriad synthetic approaches to their formation.^22–25^ Pyridopyrimidine derivatives, specifically, have garnered substantial attention due to their wide-ranging biological activities, encompassing anticancer,^26^ antiviral,^27^ antiallergic,^28^ anti-HIV,^29^ and anti-inflammatory^30^ properties. Catalysts are indispensable in certain manufacturing protocols for heterocyclic compounds. Graphene oxide (GO), acknowledged for its desirable characteristics such as a high surface area and notable adsorption capacity,^31–33^ has been extensively investigated. Nanostructured transition metal oxides, possessing elevated active surface areas, can function as catalysts in these synthetic pathways. Beyond their fundamental scientific applications, these catalytic agents find extensive employment in both technological advancements and applied research endeavors. Of late, metal oxides and supported catalysts have garnered considerable interest, largely attributable to their remarkable selectivity and efficiency in promoting organic transformations. The distinctive catalytic efficacy and inherent crystalline organization characteristic of metal oxides set them apart within this domain.^34–37^ This underscores the capacity to tailor a material's surface characteristics to achieve optimal suitability for particular applications through strategic combination of multiple metallic elements and judicious implementation of diverse processing methodologies.^38–40^ The incorporation of metal oxide catalysts within nanocomposite architectures has demonstrated remarkable effectiveness in the green synthesis of heterocyclic compounds, adhering to principles of environmental sustainability. Such organic compounds frequently exhibit antioxidant capabilities stemming from chemical configurations that facilitate the mitigation of deleterious effects, thereby counteracting the detrimental influence of free radicals.^41–43^ Furthermore, organic compounds possessing antioxidant attributes may contribute to the prevention or amelioration of specific disease states.^44–51^ Investigation into the antibacterial characteristics of synthetically derived materials constitutes another critical area of inquiry pertaining to their broader biological functionalities. Diverse bacterial strains give rise to a spectrum of diseases impacting both human and animal populations, a subset of which prove refractory to existing treatment modalities. Consequently, synthesized compounds may exhibit the dual benefit of antioxidant and antimicrobial activity. Dyes and pigments, finally, find widespread application across a multitude of industrial sectors, encompassing textiles, cosmetics, printing, pharmaceuticals, and food processing. Approximately 700 000 tons of dyes and pigments are produced annually, most of which are harmful to aquatic life. As a result, it is necessary to enhance environmentally friendly and green methods for eliminating these harmful substances from our surroundings, owing to their toxic and cancer-causing characteristics. The literature describes various chemical and physical techniques for the behaviour of dyes, including their waste. However, some drawbacks of these approaches are the high costs, the creation of dangerous by-products, and the need for significant energy. Therefore, there is a need for more sustainable methods or chemical solutions to reduce or eliminate these issues. Recent studies have concentrated on finding new and straightforward techniques for creating important heterocyclic compounds.^52–80^ This research uses an eco-friendly strategy to produce unique dipyridopyrimidines 5. The process involves a reaction with multiple components using acetylacetone 1, aldehydes 2, guanidine 3, and activated acetylenic compounds 4 in the presence of Cu/ZnO@GO as new efficient catalyst (Scheme 1).

## 2. Experimental section

### 2.1. General

The research team employed analytical reagents and solvents of exceptional purity, ensuring the stability of their physicochemical properties. To characterize the synthesized nanocatalysts and resultant dipyridopyrimidines, a Shimadzu IR-460 spectrometer was utilized. These analyses were conducted in a KBr matrix, with data acquisition performed *via* FT-IR spectroscopy. Furthermore, proton and carbon NMR spectra of the synthesized materials were acquired using a Bruker DRX-400 AVANCE spectrometer. This instrument was operated at a frequency of 400 MHz, employing CDCl_3_ as the solvent and TMS as the internal standard for the spectra of the synthesized materials. The mass spectra of these compounds were recorded on a Finnigan MAT 8430 spectrometer, utilizing an ionization energy of 70 eV. Elemental analysis of the synthesized compounds was performed using a Heraeus CHN-O-Rapid analyzer. The successful fabrication of the synthetic catalyst Cu/ZnO@GO was corroborated through a suite of spectroscopic methods, including XRD, EDX, TEM and SEM.

### 2.2. Fabrication of Cu/ZnO@GO nanocatalyst

To prepare the solution, a precise amount of 1.5 grams of zinc acetate (Zn(OAC)_2_) and 1.5 grams of CuCl_2_ were individually dissolved in 5 mL of an aqueous extract derived from the rhizome of *Petasites hybridus*. This mixture was then heated together in a round-bottom flask until reaching a temperature of 200 °C, during which it was continuously stirred for one hour. Immediately following the completion of the reaction, it was crucial to rapidly cool the mixture to ambient temperature. Once cooled, the mixture was subjected to centrifugation at 7000 rpm for approximately ten minutes to remove any unreacted organic residues. The resulting Cu/ZnO nanoparticles were subsequently isolated, washed thoroughly with a solution comprising equal parts of ethanol and water, and then allowed to dry naturally at room temperature for 24 hours. To synthesize the nanocomposite, 0.1 grams of graphene oxide (GO) and 0.1 grams of Cu/ZnO nanoparticles were combined and mixed with 10 mL of the *Petasites hybridus* rhizome extract. This suspension was then subjected to vigorous agitation at 200 °C for one hour. The Cu/ZnO@GO nanocatalyst was then washed multiple times with a 50 : 50 ethanol–water solution to ensure purity. In conclusion, the targeted nanocomposite, Cu/ZnO@GO, was successfully synthesized with a high yield, demonstrating an efficient and reproducible approach.

### 2.3. Preparation procedure for dipyridopyrimidines 5a–5j

At ambient temperature, acetylacetone 1 (1 mmol), and aldehydes 2 (2 mmol) were mixed in the presence of sodium hydride (3 mmol) and catalyst (0.02 g) for 45 min. After this time guanidine 3 (1 mmol) was introduced into the mixture and stirred for 30 minutes. We stirred the fresh concoction for 45 minutes after including the activated acetylenic compound 4 (1 mmol) into the initial mixture following a lapse of 30 minutes. The reaction was completed within a three-hour timeframe, and thin-layer chromatography (TLC) was used to monitor the reaction progress. To produce pure title compounds 5, a solid residue was isolated using filtering, subjected to cleaning with ethanol and diethyl ether, soluble in dichloromethane, and further purified using column chromatography with a 5 : 1 mixture of hexane and ethyl acetate.

#### 2.3.1 Tetramethyl 6-imino-2,10-dimethyl-10,12-dihydro-2*H*,6*H*-dipyrido[1,2-*c*:2′,1′-*f*]pyrimidine-3,4,8,9-tetracarboxylate (5a)

Yellow powder, m. p. 123–125 °C, yield: 85%. IR (KBr) (*ν*_max_/cm^−1^): 3347, 1742, 1735, 1587, 1487, 1385 and 1290 cm^−1^. ^1^H NMR (500 MHz, CDCl_3_): *δ*_ppm_ 1.25 (6H, d, ^3^*J*_HH_ = 7.2 Hz, 2CH_3_), 3.07 (1H, d, ^2^*J*_HH_ = 6.5 Hz, CH), 3.18 (1H, d, ^2^*J*_HH_ = 6.5 Hz, CH), 3.65 (6H, s, 2CH_3_O), 3.69 (2H, q, ^3^*J*_HH_ = 7.2 Hz, 2CH), 3.75 (6H, s, 2CH_3_O), 5.86 (2H, s, 2CH), 9.44 (1H, s, NH). ^13^C NMR (125.7 MHz, CDCl_3_): *δ*_ppm_ 166.7, 165.5, 154.0, 145.3, 135.4, 121.2, 120.1, 53.0, 51.6, 32.1, 28.3, 21.4. MS, *m*/*z* (%): 459 (M^+^, 10), 31 (100). Anal. calcd for C_22_H_25_N_3_O_8_ (459.16): C, 57.51; H, 5.48; N, 9.15; found: C, 57.63; H, 5.58; N, 9.28.

#### 2.3.2 Dimethyl 2,10-diethyl-6-imino-10,12-dihydro-2*H*,6*H*-dipyrido[1,2-*c*:2′,1′-*f*]pyrimidine-3,9-dicarboxylate (5b)

Yellow powder, m. p. 137–139 °C, yield: 70%. IR (KBr) (*ν*_max_/cm^−1^): 3357, 1738, 1732, 1575, 1475, 1385 and 1297. ^1^H NMR (500 MHz, CDCl_3_): *δ*_ppm_ 0.94 (6H, t, ^3^*J*_HH_ = 6.8 Hz, 2CH_3_), 1.59 (4H, m, 2CH_2_), 3.06 (2H, s, CH_2_), 3.43 (2H, t, ^3^*J*_HH_ = 6.8 Hz, 2CH), 3.70 (6H, s, 2CH_3_O), 5.74 (2H, s, 2CH), 8.24 (1H, s, 1CH), 8.67 (1H, s, NH). ^13^C NMR (125.7 MHz, CDCl_3_): *δ*_ppm_ 167.5, 151.7, 135.4, 134.3, 117.3, 109.4, 51.4, 46.7, 38.1, 27.9, 11.3. MS, *m*/*z* (%): 371 (M^+^, 10), 31 (100). Anal. calcd for C_20_H_25_N_3_O_4_ (371.18): C, 64.67; H, 6.78; N, 11.31; found: C, 64.78; H, 6.86; N, 11.45%.

#### 2.3.3 Tetramethyl 6-imino-2,10-di-*p*-tolyl-10,12-dihydro-2*H*,6*H*-dipyrido[1,2-*c*:2′,1′-*f*]pyrimidine-3,4,8,9-tetracarboxylate (5c)

Yellow powder, m. p. 165–167 °C, yield: 87%. IR (KBr) (*ν*_max_/cm^−1^): 3448, 1745, 1738, 1556, 1478, 1385 and 1280. ^1^H NMR (500 MHz, CDCl_3_): *δ*_ppm_ 2.35 (6H, s, 2Me), 3.14 (1H, d, ^2^*J*_HH_ = 6.5 Hz, CH), 3.22 (1H, d, ^2^*J*_HH_ = 6.5 Hz, CH), 3.62 (6H, s, 2MeO), 3.74 (6H, s, 2MeO), 4.74 (2H, s, 2CH), 6.12 (2H, s, 2CH), 7.15 (4H, d, ^3^*J*_HH_ = 7.6 Hz, 4CH), 7.17 (4H, d, ^3^*J*_HH_ = 7.6 Hz, 4CH), 9.44 (1H, s, NH). ^13^C NMR (125.7 MHz, CDCl_3_): *δ*_ppm_ 165.9, 165.1, 154.1, 140.8, 140.7, 137.8, 137.1, 130.1, 128.3, 128.1, 115.7, 53.0, 51.6, 38.6, 32.2, 21.0. MS, *m*/*z* (%): 611 (M^+^, 10), 31 (100). Anal. calcd for C_34_H_33_N_3_O_8_ (611.23): C, 66.77; H, 5.44; N, 6.87; found: C, 66.92; H, 5.62; N, 6.96%.

#### 2.3.4 Tetramethyl 2,10-diethyl-6-imino-10,12-dihydro-2*H*,6*H*-dipyrido[1,2-*c*:2′,1′-*f*]pyrimidine-3,4,8,9-tetracarboxylate (5d)

Yellow powder, m. p. 152–154 °C, yield: 83%. IR (KBr) (*ν*_max_/cm^−1^): 3435, 1742, 1738, 1465 and 1295. ^1^H NMR (500 MHz, CDCl_3_): *δ*_ppm_ 0.96 (6H, d, ^3^*J*_HH_ = 6.8 Hz, 2CH_3_), 1.67 (4H, m, 2CH_2_), 3.10 (1H, d, ^2^*J*_HH_ = 6.5 Hz, CH), 3.22 (1H, d, ^2^*J*_HH_ = 6.5 Hz, CH), 3.55 (2H, t, ^3^*J*_HH_ = 6.8 Hz, 2CH), 3.65 (6H, s, 2CH_3_O), 3.75 (6H, s, 2CH_3_O), 5.80 (2H, s, 2CH), 9.44 (1H, s, NH). ^13^C NMR (125.7 MHz, CDCl_3_): *δ*_ppm_ 166.8, 165.3, 154.0, 137.7, 130.2, 121.4, 111.2, 53.0, 51.6, 41.1, 32.1, 28.1, 11.3. MS, *m*/*z* (%): 487 (M^+^, 10), 31 (100). Anal. calcd for C_24_H_29_N_3_O_8_ (487.51): C, 59.13; H, 6.00; N, 8.62; found: C, 59.24; H, 6.27; N, 8.72%.

#### 2.3.5 Dimethyl 6-imino-2,10-bis(4-nitrophenyl)-10,12-dihydro-2*H*,6*H*-dipyrido[1,2-*c*:2′,1′-*f*]pyrimidine-3,9-dicarboxylate (5e)

Yellow powder, m. p. 159–161 °C, yield: 75%. IR (KBr) (*ν*_max_/cm^−1^): 3445, 1739, 1736, 1486, 1376 and 1290. ^1^H NMR (500 MHz, CDCl_3_): *δ*_ppm_ 3.08 (1H, d, ^2^*J*_HH_ = 6.5 Hz, CH), 3.17 (1H, d, ^2^*J*_HH_ = 6.5 Hz, CH), 3.67 (6H, s, 2CH_3_O), 4.80 (2H, s, 2CH), 6.03 (2H, s, 2CH), 7.54 (4H, d, ^3^*J*_HH_ = 7.6 Hz, 4CH), 8.18 (4H, d, ^3^*J*_HH_ = 7.6 Hz, 4CH), 8.25 (1H, s, 1CH), 8.67 (1H, s, NH). ^13^C NMR (125.7 MHz, CDCl_3_): *δ*_ppm_ 166.4, 152.9, 150.3, 146.6, 136.1, 135.4, 133.4, 129.3, 123.2, 109.7, 51.7, 39.8, 29.9. MS, *m*/*z* (%): 557 (M^+^, 10), 31 (100). Anal. calcd for C_28_H_23_N_5_O_8_(557.52): C, 60.32; H, 4.16; N, 12.56; found: C, 60.45; H, 4.26; N, 12.67%.

#### 2.3.6 Tetramethyl 6-imino-2,10-bis(4-methoxyphenyl)-10,12-dihydro-2*H*,6*H*-dipyrido[1,2-*c*:2′,1′-*f*]pyrimidine-3,4,8,9-tetracarboxylate (5f)

Yellow powder, m. p. 147–149 °C, yield: 90%. IR (KBr) (*ν*_max_/cm^−1^): 3442, 1740, 1738, 1578, 1475 and 1286. ^1^H NMR (500 MHz, CDCl_3_): *δ*_ppm_ 3.14 (1H, d, ^2^*J*_HH_ = 6.5 Hz, CH), 3.21 (1H, d, ^2^*J*_HH_ = 6.5 Hz, CH), 3.62 (6H, s, 2CH_3_O), 3.68 (6H, s, 2CH_3_O), 3.78 (6H, s, 2CH_3_O), 4.73 (2H, s, CH_2_), 6.03 (2H, s, 2CH), 6.86 (4H, d, ^3^*J*_HH_ = 7.6 Hz, 4CH), 7.11 (4H, d, ^3^*J*_HH_ = 7.6 Hz, 4CH), 9.44 (1H, s, NH). ^13^C NMR (125.7 MHz, CDCl_3_): *δ*_ppm_ 165.9, 165.1, 159.0, 154.1, 140.7, 137.7, 136.2, 129.5, 121.0, 115.6, 114.4, 55.3, 53.0, 51.6, 38.6, 32.2. MS, *m*/*z* (%): 634 (M^+^, 10), 31 (100). Anal. calcd for C_34_H_33_N_3_O_10_ (634.65): C, 63.45; H, 5.17; N, 6.53; found: C, 63.58; H, 5.26; N, 6.68%.

#### 2.3.7 Tetramethyl 6-imino-2,10-bis(4-nitrophenyl)-10,12-dihydro-2*H*,6*H*-dipyrido[1,2-*c*:2′,1′-*f*]pyrimidine-3,4,8,9-tetracarboxylate (5g)

Yellow powder, m. p. 178–179 °C, yield: 75%. IR (KBr) (*ν*_max_/cm^−1^): 3358, 1738, 1735, 1487 and 1268. ^1^H NMR (500 MHz, CDCl_3_): *δ*_ppm_ 3.15 (1H, d, ^2^*J*_HH_ = 6.7 Hz, CH), 3.20 (1H, d, ^2^*J*_HH_ = 6.7 Hz, CH), 3.63 (6H, s, 2CH_3_O), 3.74 (6H, s, 2CH_3_O), 4.79 (2H, s, 2CH), 5.98 (2H, s, 2CH), 7.52 (4H, d, ^3^*J*_HH_ = 7.6 Hz, 4CH), 8.16 (4H, d, ^3^*J*_HH_ = 7.6 Hz, 4CH), 9.45 (1H, s, NH). ^13^C NMR (125.7 MHz, CDCl_3_): *δ*_ppm_ 165.9, 165.1, 154.1, 149.5, 146.6, 140.7, 137.1, 129.2, 124.4, 120.9, 115.6, 53.0, 51.6, 38.6, 32.2. MS, *m*/*z* (%): 802 (M^+^, 10), 45 (100). Anal. calcd for C_32_H_27_N_5_O_12_ (673.59): C, 57.06; H, 4.04; N, 10.40; found: C, 57.22; H, 4.18; N, 10.52%.

#### 2.3.8 Dimethyl 6-imino-2,10-di-*p*-tolyl-10,12-dihydro-2*H*,6*H*-dipyrido[1,2-*c*:2′,1′-*f*]pyrimidine-3,9-dicarboxylate (5h)

Pale yellow powder, m. p. 139–141 °C, yield: 90%. IR (KBr) (*ν*_max_/cm^−1^): 3452, 1742, 1739 and 1487. ^1^H NMR (500 MHz, CDCl_3_): *δ*_ppm_ 2.35 (6H, s, 2Me), 3.09 (1H, d, ^2^*J*_HH_ = 6.7 Hz, CH), 3.17 (1H, d, ^2^*J*_HH_ = 6.7 Hz, CH), 3.67 (6H, s, 2CH_3_O), 4.80 (2H, s, CH_2_), 6.03 (2H, s, 2CH), 7.14 (4H, d, ^3^*J*_HH_ = 7.6 Hz, 4CH), 7.17 (4H, d, ^3^*J*_HH_ = 7.6 Hz, 4CH), 8.25 (1H, s, 1CH), 9.44 (1H, s, NH). ^13^C NMR (125.7 MHz, CDCl_3_): *δ*_ppm_ 166.4, 151.9, 140.6, 137.8, 136.1, 134.4, 134.2, 130.1, 128.5, 122.9, 109.7, 51.6, 39.8, 21.1. MS, *m*/*z* (%): 495 (M^+^, 10), 31 (100). Anal. calcd for C_30_H_29_N_3_O_4_ (495.58): C, 72.71; H, 5.90; N, 8.48; found: C, 72.83; H, 5.98; N, 8.56%.

#### 2.3.9 Diethyl 6-imino-2,10-dimethyl-10,12-dihydro-2*H*,6*H*-dipyrido[1,2-*c*:2′,1′-*f*]pyrimidine-3,9-dicarboxylate (5i)

Pale yellow powder, m. p. 133–135 °C, yield: 85%. IR (KBr) (*ν*_max_/cm^−1^): 1739, 1737, 1695, 1576, 1368 and 1284. ^1^H NMR (500 MHz, CDCl_3_): *δ*_ppm_ 1.22 (6H, t, ^3^*J*_HH_ = 7.3 Hz, 2CH_3_), 1.27 (6H, d, ^3^*J*_HH_ = 6.8 Hz, 2CH_3_), 2.97 (1H, d, ^2^*J*_HH_ = 6.7 Hz, CH), 3.05 (1H, d, ^2^*J*_HH_ = 6.7 Hz, CH), 3.65 (2H, q, ^3^*J*_HH_ = 7.2 Hz, 2CH), 4.21 (4H, q, ^3^*J*_HH_ = 7.2 Hz, 2CH_2_O), 5.80 (2H, s, CH), 8.23 (1H, s, CH), 8.67 (1H, s, NH). ^13^C NMR (125.7 MHz, CDCl_3_): *δ*_ppm_ 167.3, 151.6, 140.8, 134.7, 126.0, 112.2, 61.8, 49.3, 26.8, 19.6, 14.1. MS, *m*/*z* (%): 890 (M^+^, 10), 45 (100). Anal. calcd for C_20_H_25_N_3_O_4_ (371.44): C, 64.67; H, 6.78; N, 11.31; found: C, 64.85; H, 6.86; N, 11.45%.

#### 2.3.10 Diethyl 6-imino-2,10-bis(4-methoxyphenyl)-10,12-dihydro-2*H*,6*H*-dipyrido[1,2-*c*:2′,1′-*f*]pyrimidine-3,9-dicarboxylate (5j)

Pale yellow powder, m. p. 151–153 °C, yield: 90%. IR (KBr) (*ν*_max_/cm^−1^): 1742, 1739, 1587, 1378 and 1292. ^1^H NMR (500 MHz, CDCl_3_): *δ*_ppm_ 1.22 (6H, t, ^3^*J*_HH_ = 7.3 Hz, 2CH_3_), 3.08 (1H, d, ^2^*J*_HH_ = 6.2 Hz, CH), 3.17 (1H, d, ^2^*J*_HH_ = 7.3 Hz, CH), 3.78 (6H, s, 2OCH_3_), 4.12 (4H, q, ^3^*J*_HH_ = 7.3 Hz, 2CH_2_O), 4.78 (2H, s, 2CH), 6.04 (2H, s, 2CH), 6.86 (4H, d, ^3^*J*_HH_ = 7.6 Hz, 4CH), 7.18 (4H, d, ^3^*J*_HH_ = 7.6 Hz, 4CH), 8.15 (1H, s, CH), 8.67 (1H, s, NH). ^13^C NMR (125.7 MHz, CDCl_3_): *δ*_ppm_ 166.6, 159.0, 151.9, 140.8, 136.1, 135.4, 129.6, 123.1, 114.4, 110.1, 61.7, 55.3, 49.8, 43.4, 14.1. MS, *m*/*z* (%): 555 (M^+^, 10), 45 (100). Anal. calcd for C_32_H_33_N_3_O_6_ (890.94): C, 69.17; H, 5.99; N, 7.56; found: C, 69.32; H, 6.12; N, 7.72%.

### 2.4. Evaluation of antioxidant property *via* DPPH

In this investigation, the antioxidant capabilities of a series of synthesized dipyridopyrimidines (specifically compounds 5a–5d) were assessed utilizing the DPPH free radical scavenging assay, adhering to methodologies established by Shimada *et al.*^81^ Dipyridopyrimidines 5a–5d were examined across a concentration gradient of 200 to 1000 ppm, consistent with the Shimada protocol. Subsequently, an equal volume of a methanolic DPPH solution (1 mmol L^−1^) was introduced to the dipyridopyrimidine solution. Post-incubation at ambient temperature in darkness for a duration of 30 minutes with agitation, the absorbance of the resulting mixture was quantified spectrophotometrically at a wavelength of 517 nm. To contextualize the antioxidant activity of the synthesized dipyridopyrimidines 5a–5d, their performance was benchmarked against established antioxidants butylated hydroxytoluene (BHT) and 2-*tert*-butylhydroquinone (TBHQ). Control measurements utilized methanol (3 mL) *in lieu* of the synthesized compounds. Finally, the percentage of DPPH radical scavenging inhibition was calculated according to the formula articulated by Yen and Duh.^82^

### 2.5. Evaluating FRAP process of dipyridopyrimidines antioxidant activity

Employing the DPPH free radical assay, in conjunction with methodologies established by Yildirim *et al.*,^83^ the present study delves into the antioxidant properties exhibited by a series of newly synthesized dipyridopyrimidines, specifically compounds 5a–5d. Dipyridopyrimidines 5a–5d were evaluated at concentrations ranging from 200 to 1000 ppm, adhering to the parameters outlined in the Shimada method. Subsequently, an aliquot of the dipyridopyrimidine solution was combined with an equivalent volume of a 1 mmol per L DPPH solution prepared in methanol. The resulting mixture, after incubation in darkness for 30 minutes at ambient temperature, underwent spectrophotometric analysis at 517 nm to determine its absorbance. In order to ascertain the antioxidant efficacy of dipyridopyrimidines 5a–5d, a comparative analysis was undertaken utilizing TBHQ and BHT as reference standards, with methanol (3 mL) serving as the blank control *in lieu* of the synthesized compounds.

### 2.6. Examining antibacterial activity of the prepared dipyridopyrimidines

Employing the disk diffusion method, a Persian-type culture collection (PTCC) comprising both Gram-positive and Gram-negative bacteria was established. This investigation aimed to ascertain the antibacterial activity of selected dipyridopyrimidines, namely compounds 5a, 5d, 5e, and 5h. To assess the aforementioned dipyridopyrimidines' efficacy, bacterial cultures were cultivated at 37 °C for a period of 16 to 24 hours, adhering to the McFarland Standard No. 0.5 preparation protocol. Streptomycin and gentamicin served as the principal antimicrobial agents for comparative analysis. A bacterial suspension was prepared by inoculating a disinfected swab onto Mueller Hinton agar, conforming to the McFarland Standard No. 0.5 (1.5 × 10^8^ CFU mL^−1^). Subsequent to the application of dipyridopyrimidines 5a, 5d, 5e, and 5h (at a concentration of 25 μg mL^−1^) onto sterile blank disks to evaluate their antibacterial characteristics, the specimens underwent incubation at 37 °C for 24 hours. The resulting zones of inhibition were then measured and contrasted with those observed in the control group.

### 2.7. Catalytic performance of the Cu/ZnO@GO in organic pollutants such as 4-NP reduction

In a typical experimental run, a mixture comprising 25 mL of 4-nitrophenol solution (2.5 mM) and 5.0 mg of Cu/ZnO@GO catalyst was prepared within a beaker and left undisturbed at ambient temperature for a period of 2 minutes. Subsequently, the reaction was initiated by the introduction of 25 mL of a freshly prepared 0.25 M NaBH_4_ solution into the same beaker. Upon addition of the NaBH_4_ solution, an immediate visual transformation occurred, with the solution's color changing almost instantaneously from a pale yellow to a more vibrant lemon-yellow shade. This mixture underwent continuous stirring until complete decolorization was observed. Post-decolorization, a 1 mL aliquot of the solution was subjected to a 25-fold dilution to enable subsequent absorbance measurements *via* UV-Vis spectroscopy at predetermined time points. The temporal variation in 4-nitrophenol concentration was monitored by recording UV-Vis absorption spectra over a wavelength range of 200 to 700 nm, maintaining room temperature throughout the analysis. To assess the recyclability of the catalyst, it was recovered *via* filtration, thoroughly rinsed with ethanol, dried to remove residual solvent, and then subjected to repeated catalytic cycles.

## 3. Results and discussion

**Fig. 1:**
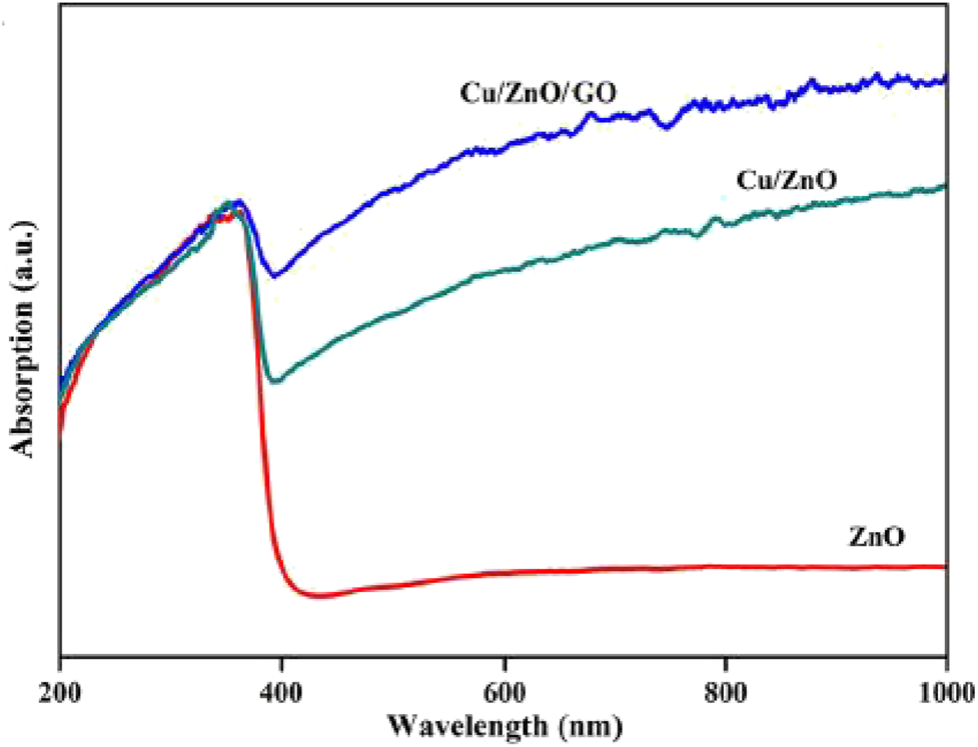
FT-IR (KBr) spectra of Cu/ZnO@GO.

The current investigation focused on the synthesis of a series of dipyridopyrimidines 5a–5j*via* a four-component reaction involving acetylacetone 1, aldehydes 2, guanidine 3, and activated acetylenic compounds 4, conducted in aqueous media at ambient temperature (Scheme 1). The reactions, occurring in an aqueous milieu at standard room temperature (Scheme 1), employed Cu/ZnO@GO as a recoverable nanocatalyst. High efficiency characterized the production of the targeted compounds. The catalytic efficacy of the Cu/ZnO@GO nanocatalyst was evaluated through its capacity to facilitate dipyridopyrimidine formation. The generated catalyst, Cu/ZnO@GO, contains Lewis acid sites (Cu and Zn) that help in the nucleophilic attack on active carbonyl groups, along with a Lewis base site (O) which effectively eliminates acid hydrogen from the reactants, leading to the formation of intermediates. Additionally, metal oxides show high crystallinity, which is vital for their catalytic effectiveness, and the synergy of two or more metals along with different processing techniques enhances the surface characteristics of the materials, making them suitable for targeted applications. The newly created Cu/ZnO@GO nanocatalyst was thoroughly characterized using various analytical methods. Characterization techniques employed included X-ray diffraction (XRD), energy dispersive X-ray spectrometry (EDS), Fourier-transform infrared spectroscopy (FT-IR), transmission electron microscopy (TEM), and field emission scanning electron microscopy (FE-SEM). The FT-IR spectra of ZnO, Cu@ZnO, and Cu/ZnO@GO samples are presented in (Fig. 1). Prominent peaks observed at 3330 cm^−1^ and 3337 cm^−1^ correspond to the H–O–H stretching vibrations associated with water molecules incorporated within the synthesized particles. Spectral signatures at 1020 cm^−1^ and 652.17 cm^−1^ are indicative of metal–oxide–metal vibrational modes, specifically those associated with Zn–O–Zn and Zn–O–Cu stretching. Vibrations detected at 465 cm^−1^ and 455 cm^−1^ are ascribed to the [M–O] vibrations of the metal oxides, corresponding to Zn–O.

**Fig. 2:**
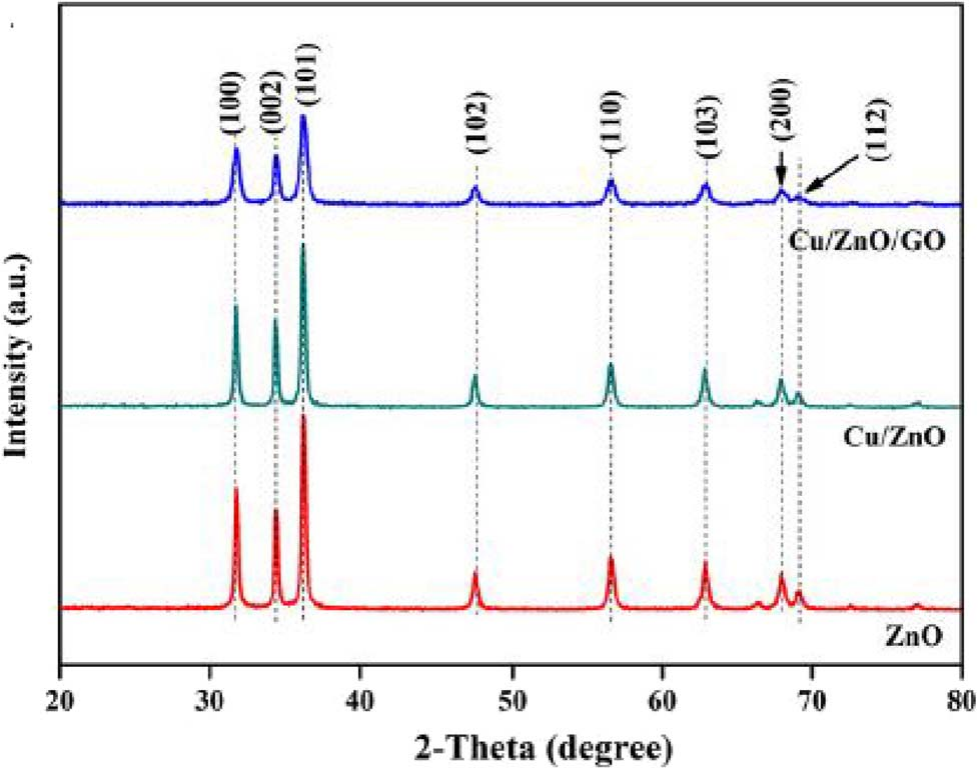
X-ray diffraction patterns of Cu/ZnO@GO.

The X-ray diffraction patterns for the Cu/ZnO@GO nanocatalyst are shown in (Fig. 2). The lack of a clear structure in the Cu/ZnO@GO particles can be linked to the gradual layering of Cu nanoparticles and ZnO on the graphene oxide (GO) surface. The strong peaks observed further support the small size and nanoscale characteristics of the particles. Specific peaks in the X-ray diffraction (XRD) pattern of the nanocatalyst relate to the ZnO and Cu nanoparticles. The ZnO peaks are correctly matched to the hexagonal phase with a wurtzite structure, as specified in JCPDS No. 01-082-9744. The peaks for Cu match the standard powder diffraction card noted as JCPDS No. 01-087-0717. As shown in Fig. 2, adding Cu leads to a decrease in the peak intensities for the main planes linked to ZnO (100), (002), and (101).

**Fig. 3:**
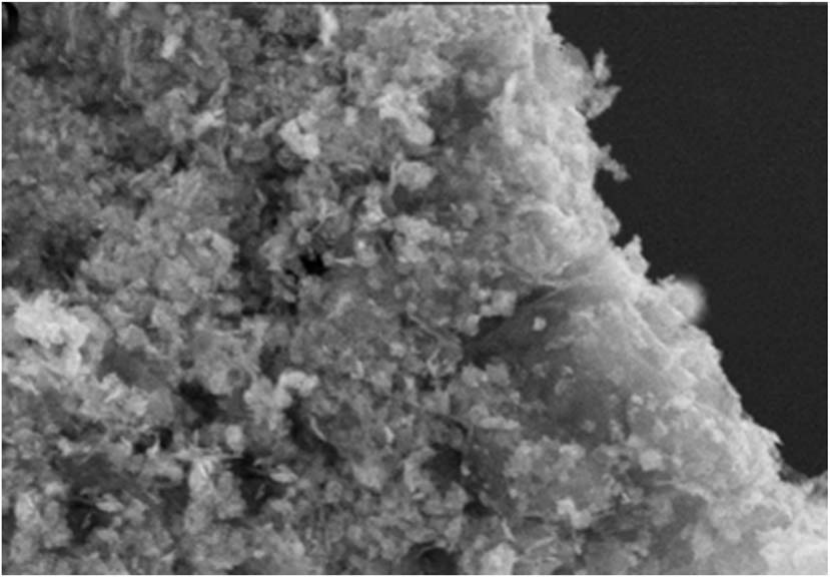
The FESEM analysis of Cu/ZnO@GO.

Cu/ZnO@GO FESEM images are shown in (Fig. 3), providing tangible evidence of the nanocatalyst effective production.

**Fig. 4:**
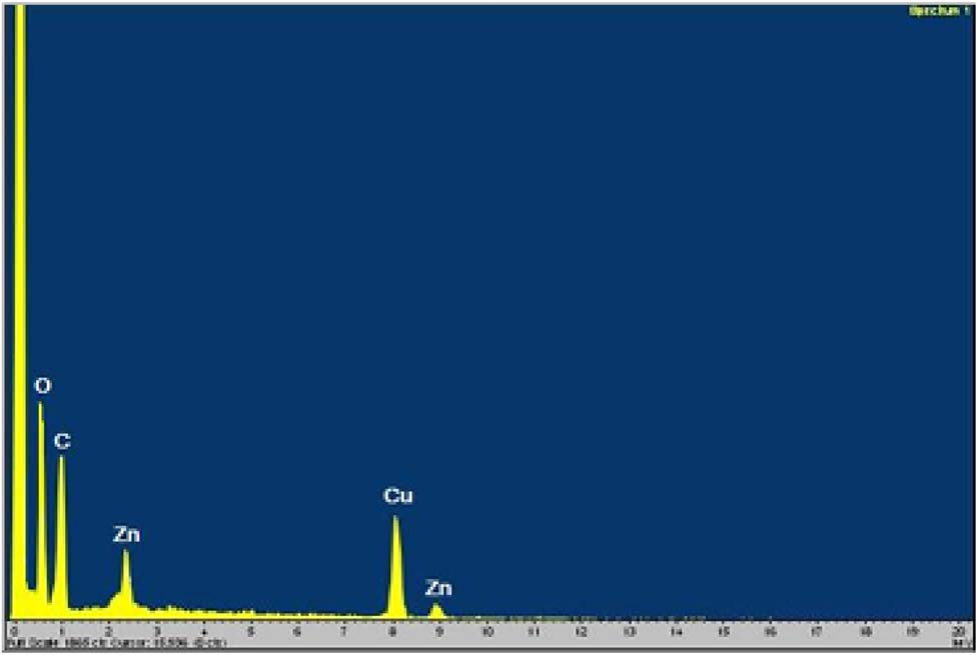
The EDX analysis of Cu/ZnO@GO.

Utilizing energy-dispersive X-ray (EDX) spectroscopic analysis, the relative abundance and chemical composition of the various constituents within the nanocatalyst were determined (see Fig. 4). The results from this analysis strongly suggest that the synthesis of Cu@ZnO@GO was successfully achieved. As illustrated in Fig. 4, the presence of copper (Cu), zinc (Zn), oxygen (O), and carbon (C) elements was identified.

**Fig. 5:**
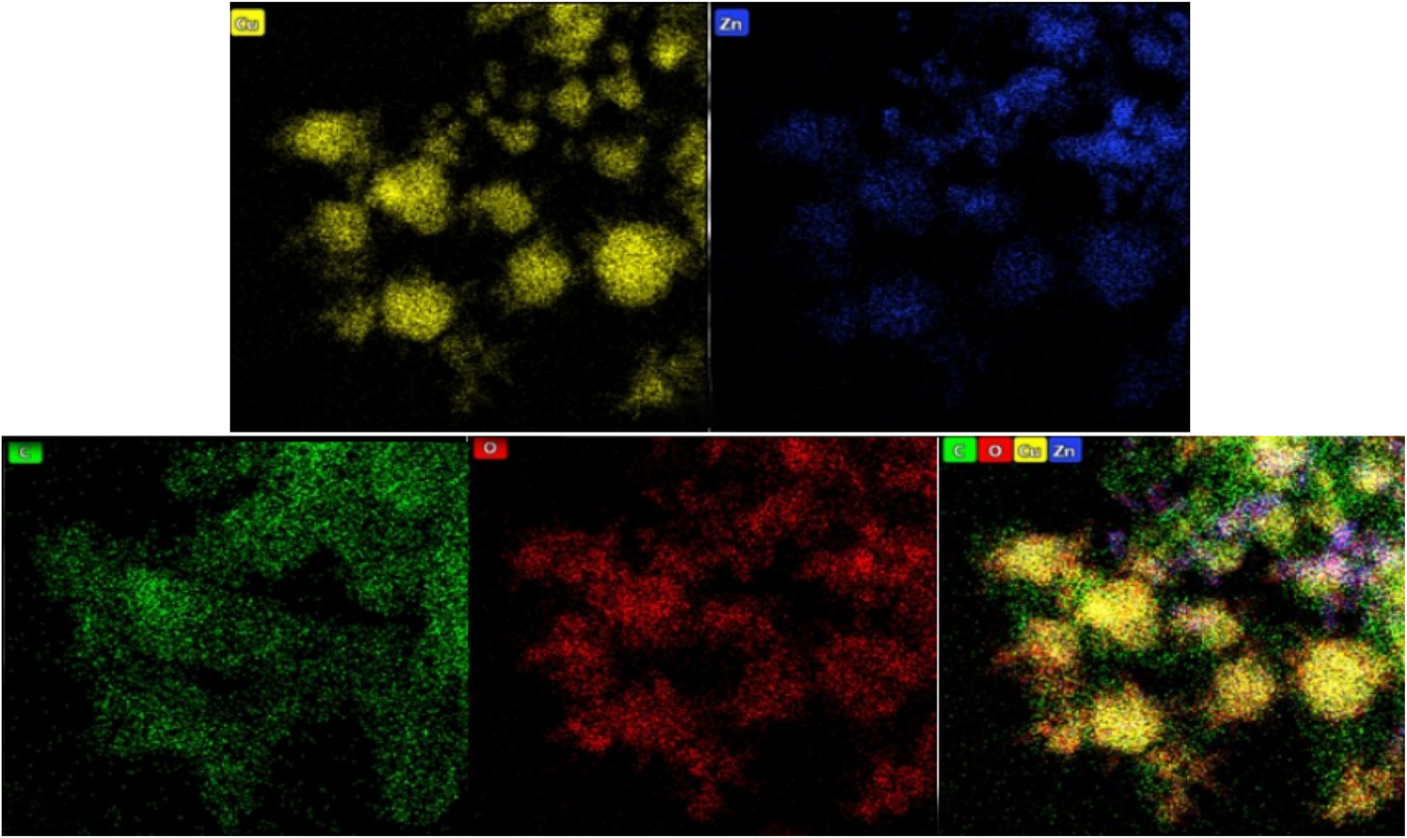
EDS mapping analysis of Cu/ZnO@GO.

Furthermore, elemental mapping was performed to examine the spatial distribution of these elements within the Cu@ZnO@GO nanocatalyst (refer to Fig. 5). This analysis corroborated the previous findings, confirming with high clarity that Cu, Zn, O, and C were distributed throughout the material with a suitable degree of dispersity. Consequently, the outcomes derived from the elemental mapping convincingly validated the initial EDX analysis, thereby reinforcing the reliability of the elemental composition data.

**Fig. 6:**
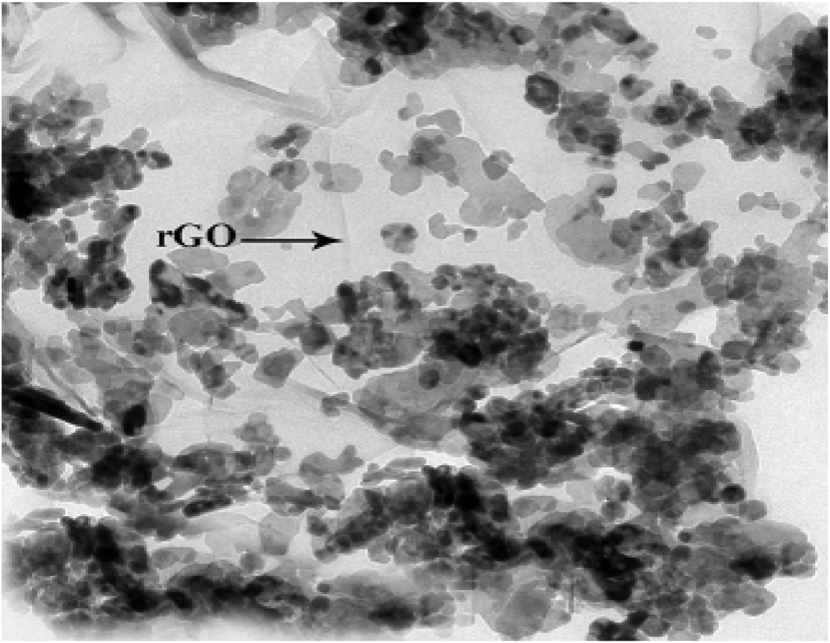
TEM images analysis of Cu/ZnO@GO.

To conduct a more detailed analysis of the morphological characteristics of the Cu@ZnO/GO nanocatalyst, Transmission Electron Microscopy (TEM) was employed to examine the samples, as illustrated in (Fig. 6). The results indicate that the Cu@ZnO nanoparticles are effectively anchored onto the GO support.

In the field of organic chemistry, optimizing reaction conditions is essential because it significantly influences the effectiveness and outcome of the experiments. To achieve this, we initially chose the experimental model including acetylacetone 1, acetaldehyde 2a, guanidine 3, and dimethyl acetylenedicarboxylate 4a (refer to Table 1). Even after a period of 10 hours, the amount of compound 5a without any catalyst is trace (entry 1, Table 1). To support this observation, we introduced a catalyst with 0.01 g of Cu nanoparticles into the reaction mixture. The successful synthesis of dipyridopyrimidine 5a, as detailed in entry 3 of Table 1, was achieved with notable efficacy (45%) within a 3-hour reaction period. Consequently, the presence of a catalyst proved indispensable for facilitating favorable reaction outcomes. An extensive evaluation was undertaken involving a range of nanocatalysts, including copper nanoparticles (NPs), zinc oxide (ZnO), and related materials. Specifically, ZnO@MWCNTs and ZnO/Cu nanoparticles (NPs) were explored to ascertain the optimal catalyst for the model reaction. Cu/ZnO@GO nanocatalyst was ultimately selected for the synthesis of dipyridopyrimidine 5a, affording an improvement in product yield. Modulation of the Cu/ZnO@GO quantity within the range of 0.01–0.03 g led to observable variations in reaction efficiency. A quantity of 0.02 g of Cu/ZnO@GO was employed in the synthesis of dipyridopyrimidine 5a, yielding a considerable product amount, as documented in entry 11 of Table 1. As referenced in entry 11 of Table 1, compound 5a was generated with an 85% yield following a 3-hour reaction. The application of nanocatalysts in the production of dipyridopyrimidine derivatives necessitates both Lewis acid and Lewis base functionality. The metals copper (Cu) and zinc (Zn) are capable of acting as Lewis acids, thereby activating the carbonyl group and rendering it more susceptible to nucleophilic attack.

**Table 1:** Determining the best conditions, including catalyst, amount of catalyst and temperature for the synthesis of 5a

Entry	Catalyst	Temp. (°C)	Catalyst (g)	TON	TOF	Time (h)	aYield%
1	None	r. t.	—	—	—	10	Trace
2	None	100	—	—	—	10	Trace
3	Cu NPs	r. t.	0.01	45	15	3	45
4	ZnO NPs	r. t.	0.015	37.3	12.4	3	56
5	ZnO NPs	r. t.	0.02	32.5	10.8	3	65
6	ZnO NPs	r. t.	0.025	26	8.67	3	65
7	ZnO@MWCNTs	r. t.	0.02	35	11.67	3	70
8	MWCNTs	r. t.	0.02	12.5	4.17	3	25
9	Cu@ MWCNTs	r. t.	0.02	27.5	9.17	3	55
10	Cu/ZnO	r. t.	0.02	39	13	3	78
11	Cu/ZnO@GO	r. t.	0.02	42.5	14.17	3	85
12	Cu/ZnO@GO	100	0.02	42.5	14.18	3	85

a Isolated yields.

The sample response was optimized by raising the temperature to 100 °C. However, the efficacy of dipyridopyrimidines 5a remained unchanged as a consequence of this adjustment (entry 2 and entry 12, Table 1). Furthermore, this investigation aimed to examine the impact of solvents on the synthesis of compound 5a when Cu/ZnO@GO (0.02 g) was present. Water is by far the best solvent for promoting the reaction, according to the data in Table 2.

**Table 2:** Determining the best solvent for generation of 5a

Entry	Solvent	Time (h)	aYield%
1	EtOH	15	78
2	CH_2_Cl_2_	8	75
3	CHCl_3_	5	75
**4**	**H**_**2**_ **O**	**3**	**85**
5	Solvent-free	8	60
6	DMF	12	45
7	Toluene	12	75
8	CH_3_CN	5	87

a Isolated yields.

Tables 1 and 2 show how effective Cu/ZnO@GO (0.02 g) is as an organometallic catalyst for creating dipyridopyrimidines 5. The catalyst works best when utilized in an aqueous environment and at normal room temperature. A key part of generating organic compounds involves the recycling of a catalyst. Water is vital for life on Earth and serves as the preferred solvent in nature's processes. In “on-water” reactions, water does not act as a solvent but instead supports reactants on its surface in an emulsion. On-water reactions refer to a category of organic chemical reactions occurring as emulsions in water. This concept has been recognized since 2005 when researchers from K. Barry Sharpless's team conducted a detailed study on this topic.^84^ Unlike in-water reactions, lipophilic substances cluster together to create a watery suspension (on water). When surfactants form a self-organized aggregate to hold lipophilic reactants, it is possible to differentiate reactions that take place inside this aggregate. In these cases, hydrogen bonding can play a role in enhancing reactions in water. By increasing the number of hydrogen bonds through a larger interfacial area, the speed of the reaction should also improve. Indeed, the same researchers showed through optical measurements that the stirring rate is linked to the interfacial area in the reaction between cyclopentadiene and methyl acrylate in water. They found that as these values increased, so did the conversion rates.^85,86^Table 3 demonstrates the successful use of the prepared nanocatalyst in the research to produce dipyridopyrimidines 5a across four different trials. The nanocatalyst was removed from the reaction mixture through filtration and was set up for reuse. It should then be rinsed with water and left to dry in the air for an entire day at room temperature. After that, it can be applied for future uses.

**Table 3:** Reusability of catalyst for synthesis of compound 5a

Run	% yield^a^
1st	85
2nd	85
3rd	85
4th	80
5th	78

a Isolated yields.

**Fig. 7:**
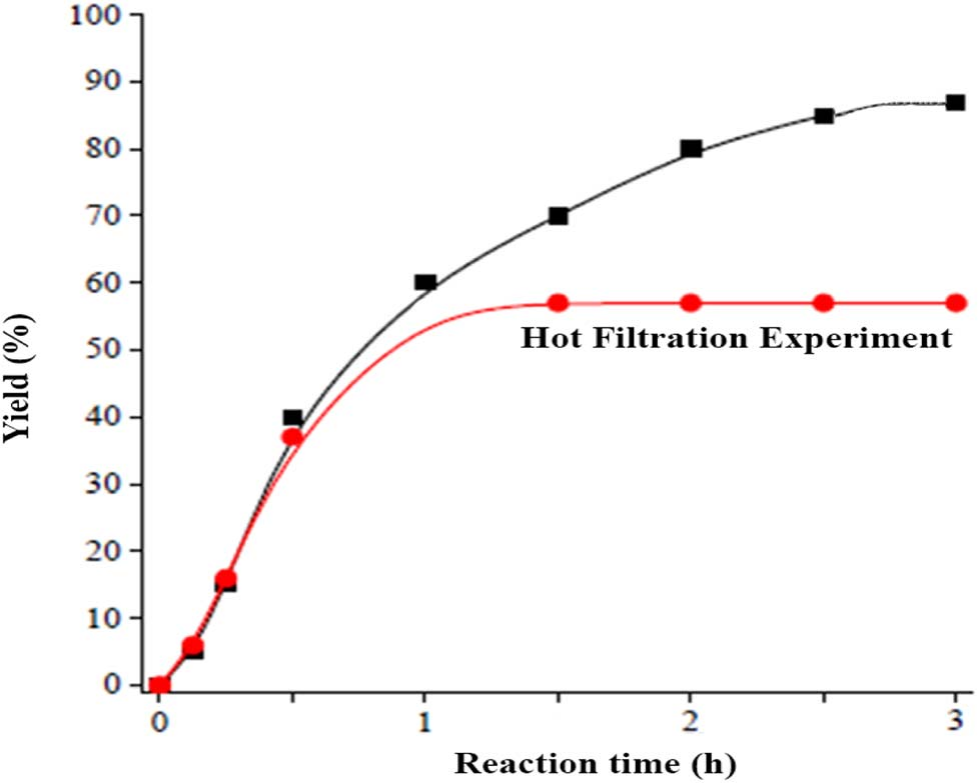
Hot filtration of Cu/ZnO@GO.

Through close observation, the process of measuring catalyst leaching can be done by using hot filtration. It is best to take out the catalyst from the mixture once the reaction's conversion rate hits 54%. Catalyst leaching occurs if the reaction keeps going. On the other hand, if the reaction stops, it can be said that leaching is not a concern (Fig. 7).

**Scheme 2:**
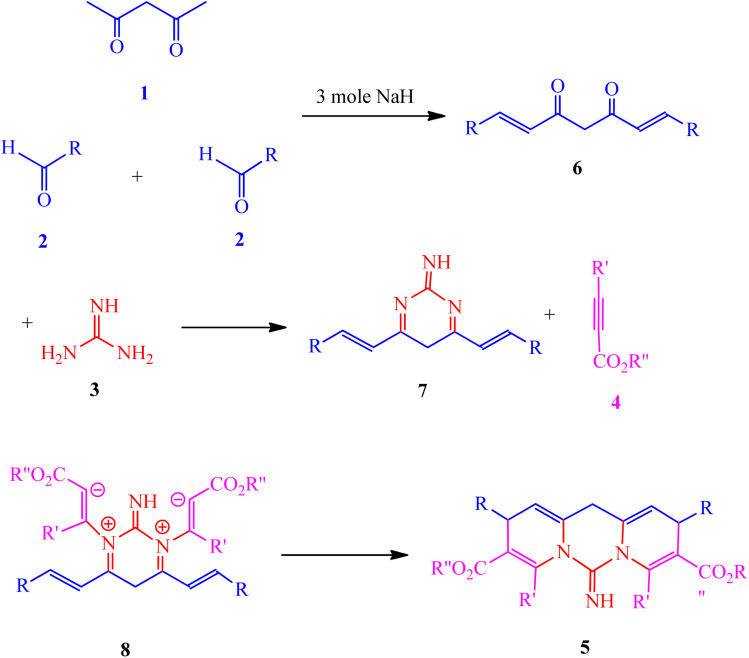
Proposed mechanism for the formation of 5.

To ascertain the precise chemical composition of the synthesized dipyridopyrimidine compound 5, a comprehensive suite of analytical methodologies was implemented. These included proton nuclear magnetic resonance (^1^HNMR) spectroscopy, carbon-13 nuclear magnetic resonance (^13^CNMR) spectroscopy, infrared (IR) spectroscopy, elemental analysis, and mass spectrometry. Analysis of the ^1^HNMR spectra of dipyridopyrimidines revealed that compound 5a exhibited a doublet signal at 1.25 ppm, a characteristic feature indicative of protons within two methyl (CH_3_) groups. A two-doublet peak was also observed at 3.07 and 3.18 ppm, corresponding to two methylene protons diastereotopic. Furthermore, two singlet peaks at 3.65 and 3.75 ppm were recorded, attributable to methoxy groups, alongside another singlet at 9.44 ppm, indicative of NH protons. Examination of the ^13^CNMR spectra of compound 5a led to the identification of a single resonance for the C=NH group, with chemical shifts registered at 153.9 ppm and C=O groups at 166.7 and 165.5 ppm. Moreover, infrared (IR) spectroscopy provided valuable corroboration regarding the presence of specific functional groups inherent to the molecular structure of the compound. Scheme 2 provides a clear illustration of the most efficacious procedure employed for the generation of the synthesized compounds 5. The current experimental investigation involves the reaction of unsaturated acetylacetone 1, aldehydes 2, and Cu/ZnO@GO (0.02 g), yielding intermediate 6, which subsequently reacts with guanidine 3 to produce intermediate 7. Intermediate 7 and activated acetylenic compounds 4 react and produced intermediate 8. This intermediate 8 then undergoes an intermolecular cyclization reaction, ultimately resulting in the formation of compound 5.

**Scheme 3:**
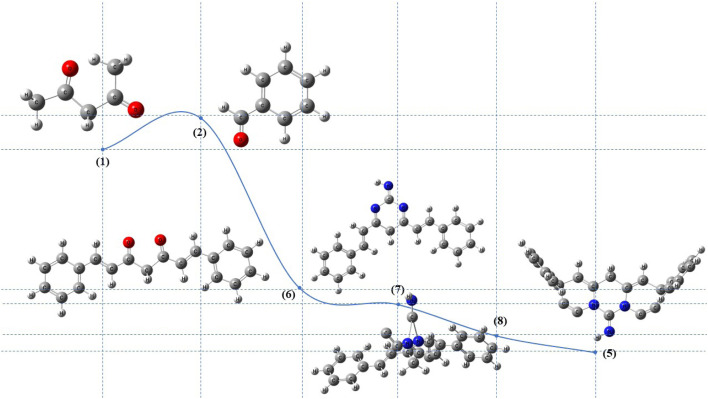
Profile of the activated intermediates for the formation of 5 that calculated by B3LYP/6-311G(d,P) level of theory.

The theoretical investigation of proposed reaction mechanism depicted in Scheme 3, illustrates the reaction pathway by charting the total energy landscape between reactants and products. The pathway suggests a mechanism proceeding *via* the formation of six distinct intermediates. These activated intermediates were subjected to optimization at the B3LYP/6-311G(d,p) level of theory, corroborating the proposed mechanism through an assessment of the intermediates' stability and the feasibility of their formation.^87–89^

### 3.1. Catalytic performance of the Cu/ZnO@GO in 4-NP reduction

**Fig. 8:**
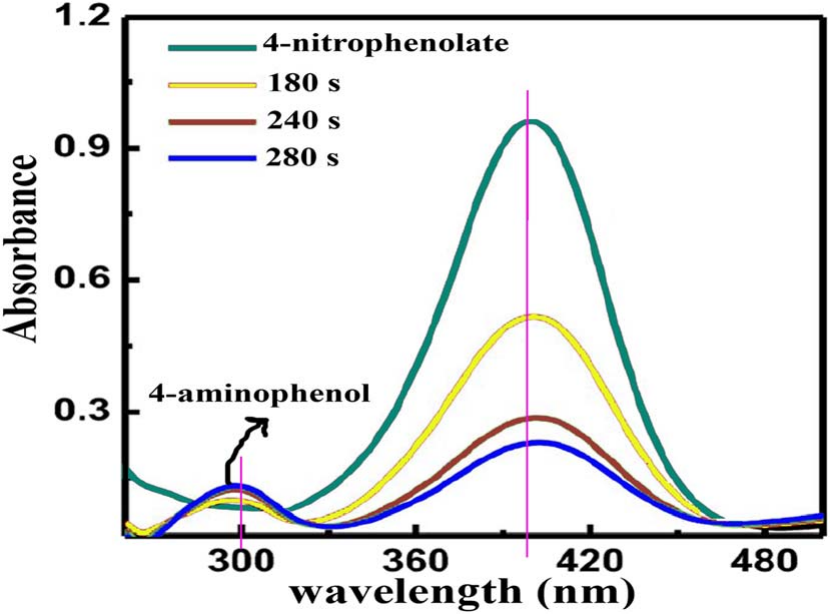
UV-vis spectrum of reduction of 4-nitrophenol and photocatalytic reduction of the 4-NP.

To scrutinize the catalytic behavior of synthesized Cu/ZnO@GO within an aqueous medium at ambient temperature, 4-nitrophenol was selected as a model substrate in this investigation. The progression of the reduction processes involving the dye was monitored *via* alterations in the UV-vis absorption spectrum of the reaction mixture, maintained at room temperature, following the removal of the catalyst through centrifugation. Initially, the transformation of 4-nitrophenol into 4-aminophenol (4-AP) was examined, utilizing Cu/ZnO@GO as the catalyst in a model reaction. Control experiments, wherein the catalyst was excluded but NaBH_4_ was present, yielded negligible conversion, thus underscoring the indispensable role of Cu/ZnO@GO in facilitating the reduction of 4-nitrophenol to 4-aminophenol. The reaction's advancement was quantified using UV-vis spectroscopy, and the resultant data is presented graphically in (Fig. 8). Characteristically, 4-nitrophenol exhibited an absorption maximum at 317 nm in aqueous solution. Upon the introduction of freshly prepared aqueous NaBH_4_ solution, a bathochromic shift in the 4-nitrophenol absorption band from 317 nm to 400 nm was observed, concomitant with a noticeable color transition from light yellow to bright yellow, indicative of 4-nitrophenolate ion formation. Notably, in the absence of Cu/ZnO@GO, the absorption peak at 400 nm remained unaltered even after a 15-hour period. This observation suggests that NaBH_4_, despite its potency as a reducing agent, is incapable of reducing the 4-nitrophenolate ion in the absence of the catalyst, even with the presence of an electron donor (BH_4_^−^) and a proton source (H_2_O) within the reaction milieu. Furthermore, the absorbance of a 4-nitrophenol solution remained constant over several hours when Cu/ZnO@GO was introduced in the absence of NaBH_4_. However, when both Cu/ZnO@GO and NaBH_4_ were present, the absorbance at 400 nm quickly dropped to nearly zero within 10 minutes, while a new peak appeared around 300 nm, attributed to 4-aminophenol, and increased gradually as the yellow color of the solution faded.

In light of these considerations, Cu/ZnO@GO was employed in this study to facilitate the photocatalytic reduction of 4-nitrophenol (4-NP). The prepared Cu/ZnO@GO composite microspheres demonstrated superior photocatalytic activity compared to their counterparts, an enhancement attributed to the synergistic advantages conferred by the incorporation of copper nanoparticles (Cu NPs), which augment light absorption. Significantly, these composite microspheres are amenable to facile removal from the reaction medium *via* external means, and subsequent reuse demonstrated only marginal diminution in catalytic efficacy. Consequently, these findings suggest that Cu/ZnO@GO composite microspheres present a viable, environmentally benign, and economically attractive approach for the elimination and photoreduction of 4-NP.

### 3.2. Assessing the antioxidant capacity of synthesized dipyridopyrimidines using the DPPH method

**Fig. 9:**
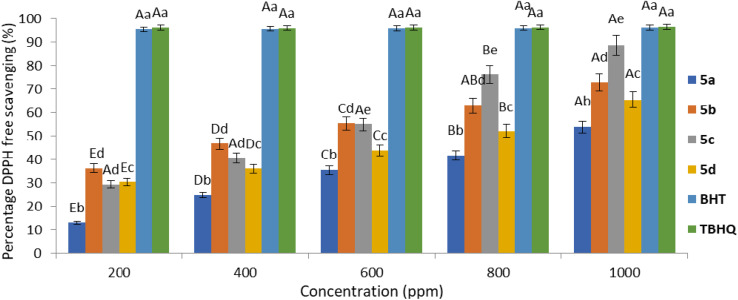
Order of antioxidant activity of 5a–5d using DPPH.

Additionally, the goal of this study was to explore the antioxidant properties of the synthesized dipyridopyrimidines. To achieve this goal, the DPPH test was used. The DPPH radical scavenging method is a common technique for measuring the antioxidant strength of synthetic substances, food products, and biological materials.^90,91^ This experiment investigates how the DPPH free radical interacts with the sample being tested. In this process, the sample has the ability to take in either an electron or a hydrogen atom from the DPPH radical. The dipyridopyrimidines showed their antioxidant abilities because they could lose an electron or a hydrogen atom in the presence of the DPPH radical. The relative antioxidant activity is assessed by how well the created dipyridopyrimidines can capture DPPH free radicals. This research involved evaluating the antioxidant properties of a set of synthetic compounds, specifically 5a–5d. We compared the antioxidant activity of these compounds with the known antioxidants BHT and TBHQ. The measurement of antioxidant activity was conducted by assessing how these compounds absorbed electrons or hydrogen using the DPPH radical method. When an electron or hydrogen atom is added to the DPPH, its absorbance at a wavelength of 517 nm decreases. The antioxidant effectiveness of dipyridopyrimidine derivatives 5a–5d was arranged in this way: TBHQ and BHT showed comparable antioxidant strengths, while 5a–5d had slightly lower capabilities, as shown in Fig. 9.

The data presented in Fig. 8 indicate notable disparities in the levels of dipyridopyrimidines in comparison to BHT and TBHQ, which are widely employed as conventional antioxidants. Within the tested group of dipyridopyrimidines, compounds 5a–5d, compound 5c displayed substantial efficacy in contrast to BHT and TBHQ.

### 3.3. Assessment of dipyridopyrimidines antioxidant activity using Fe^3+^ reducing

**Fig. 10:**
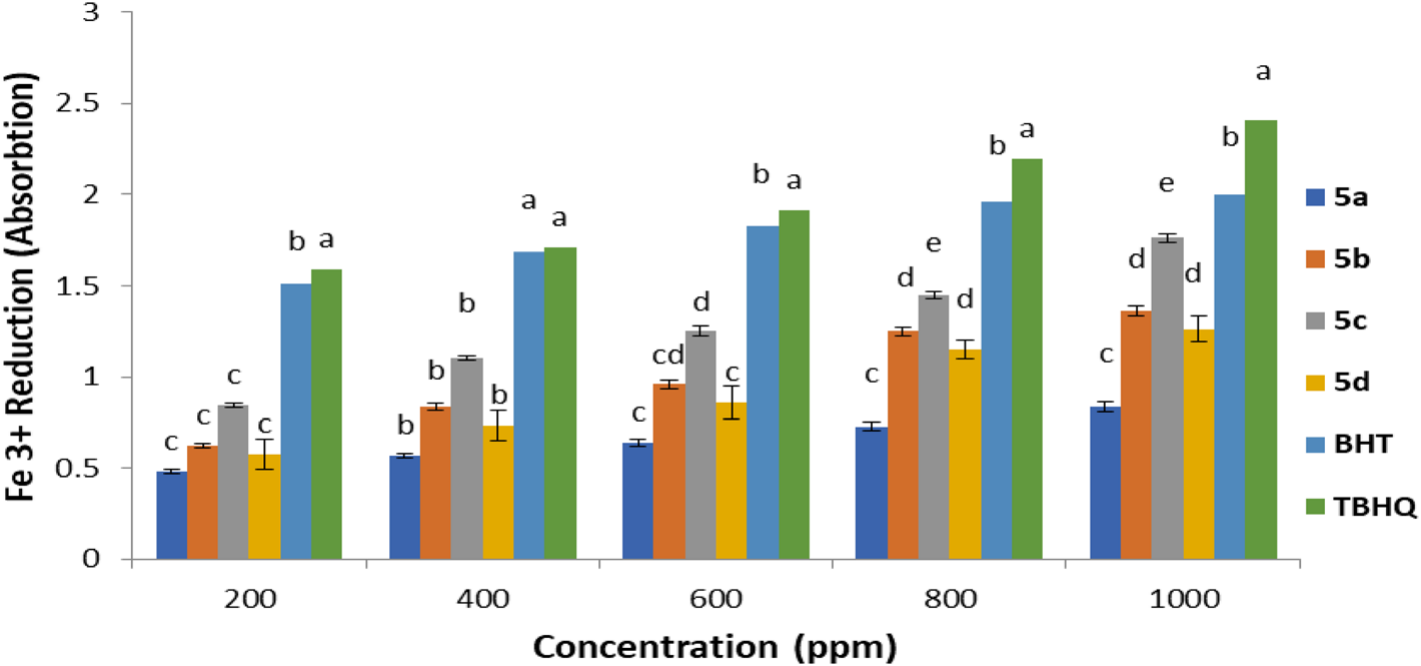
Ferric ions (Fe^3+^) decreasing antioxidant ability (FRAP) of compounds 5a–5d.

To ascertain the antioxidant efficacy of dipyridopyrimidines 5a–5d, an alternative methodological approach was implemented. It was observed that these compounds facilitated the reduction of ferric ions (Fe^3+^). The degree of this reduction was quantified by spectrophotometrically measuring the conversion of Fe^3+^/ferricyanide to Fe^2+^/ferrous at 700 nm. Notably, dipyridopyrimidine 5c exhibited a significantly more pronounced positive effect than both butylated hydroxytoluene (BHT) and tertiary butylhydroquinone (TBHQ). The relative antioxidant potencies of dipyridopyrimidines 5a–5d are presented in (Fig. 10), arrayed in the following descending order: TBHQ > BHT > 5c > 5b > 5d > 5a. It is worth noting that tertiary butylhydroquinone (TBHQ) is a structural analog of butylated hydroxytoluene (BHT).

### 3.4. Evaluation of antibacterial activity of synthesized dipyridopyrimidines

This investigation probed the antimicrobial efficacy of synthesized dipyridopyrimidines against both Gram-positive and Gram-negative bacterial strains. The performance of these newly synthesized compounds was benchmarked against that of streptomycin and gentamicin, commonly employed as conventional antibacterial agents. A detailed overview of the research outcomes is presented in Table 4. Our analysis revealed that both the specific bacterial species under consideration and the concentration of the produced chemical entities exert a considerable influence on the resulting inhibitory zone diameter. As evidenced by the data compiled in Table 4, dipyridopyrimidines designated 5a, 5d, 5e, and 5h manifested notable antimicrobial properties effective against both Gram-positive and Gram-negative bacteria. Notably, the strongest inhibition was noted against *E. coli*, a phenomenon attributable to the substantial width of the zone of inhibition exhibited.

**Table 4:** Antibacterial activity of synthesized compounds 5

Compounds	*Staphylococcus aureus* (+) PTCC 1337		*Bacillus cereus* (+) PTCC 1023		*Escherichia coli* (−) PTCC1270		*Klebsiella pneumoniae* (−) PTCC 1290	
	IZ^a^ (mm)	MIC^b^ (μg mL^−1^)	IZ (mm)	MIC (μg mL^−1^)	IZ (mm)	MIC (μg mL^−1^)	IZ (mm)	MIC (μg mL^−1^)
5a	18 ± 0.001	25	21 ± 0.001	25	22 ± 0.002	25	16 ± 0.001	25
5b	10 ± 0.002	30	8 ± 0.002	30	10 ± 0.003	30	7 ± 0.001	30
5c	17 ± 0.086	25	19 ± 0.075	25	22 ± 0.063	25	17 ± 0.054	25
5d	20 ± 0.001	20	21 ± 0.001	20	23 ± 0.024	20	18 ± 0.002	20
5e	19 ± 0.00	20	22 ± 0.003	19	21 ± 0.001	22	19 ± 0.003	22
5f	10 ± 0.001	30	9 ± 0.001	30	9 ± 0.00	30	8 ± 0.012	30
5g	10 ± 0.023	20	9 ± 0.001	20	9 ± 0.003	20	8 ± 0.003	20
5h	18 ± 0.003	25	22 ± 0.001	25	21 ± 0.001	25	18 ± 0.001	25
5i	18 ± 0.00	25	20 ± 0.003	25	21 ± 0.00	25	19 ± 0.002	25
5j	12 ± 0.001	22	10 ± 0.00	20	10 ± 0.014	20	9 ± 0.003	22
Streptomycin	19 ± 0.00	12.5	22 ± 0.00	12.5	23 ± 0.00	12.5	21 ± 0.00	12.5
Gentamicin	20 ± 0.001	12.5	24 ± 0.00	12.5	22 ± 0.00	12.5	20 ± 0.001	12.5

a Zone of inhibition in diameter in mm.

b Minimum inhibitory concentration MIC (μg mL^−1^).

## 4. Conclusion

In overview, the synthesis of the Cu/ZnO@GO nanocatalyst was successfully achieved through a method characterized by efficiency, simplicity, cost-effectiveness, and rapidity. The formation and integrity of the nanocatalyst were extensively validated using a suite of analytical techniques, including energy-dispersive X-ray spectroscopy (EDS), X-ray diffraction (XRD), scanning electron microscopy (SEM), Fourier-transform infrared spectroscopy (FT-IR), and transmission electron microscopy (TEM). Furthermore, the potential of Cu/ZnO@GO as an innovative nanocatalyst was explored in the context of synthesizing derivatives of dipyridopyrimidines. This synthetic process yielded the desired products within a timeframe of three hours, attaining yields ranging from 70% to 90%. The advantages of this protocol encompass notably brief reaction durations, high product yields, the utilization of aqueous medium as a solvent, and alignment with principles of green chemistry. In addition, two different approaches were employed to evaluate the antioxidant activity of the synthesized dipyridopyrimidines, labeled as compounds 5a–5d. The antimicrobial properties of these compounds were also assessed *via* disk diffusion assays against a spectrum of both Gram-positive and Gram-negative bacterial strains. To deepen the mechanistic understanding, density functional theory (DFT) calculations were performed, providing insights into the reaction pathway, the energetic landscape of key intermediates, and the overall plausibility of their formation.

## Author contributions

E. Ezzatzadeh conceived the original idea and was responsible for designing the research framework. N. Karami Hezarcheshmeh carried out the research. E. Ezzatzadeh analyzed the data. R. Akbari wrote the original draft. E. Ezzatzadeh reviewed the manuscript and made edits.

## Conflicts of interest

The authors declare no conflict of interest, financial or otherwise.

## Supplementary Material

RA-015-D5RA04054J-s001

## Data Availability

The data supporting this study's findings are available in this article's SI. See DOI: https://doi.org/10.1039/d5ra04054j.

## References

[cit1] Zhi S., Ma X., Zhang W. (2019). Org. Biomol. Chem..

[cit2] Ibarra I. A., Islas-Jácome A., González-Zamora E. (2018). Org. Biomol. Chem..

[cit3] Maleki A. (2018). Ultrason. Sonochem..

[cit4] Maleki A. (2014). Helv. Chim. Acta.

[cit5] Shaabani A., Maleki A. (2008). Chem. Pharm. Bull..

[cit6] Tietze L. F. , Bsasche C. and Gericke K. M., Domino Reactions in Organic Synthesis. Wiley-VCH, Weinheim, 2006

[cit7] Weber L., Illgen M., Almstetter M. (1999). Synlett.

[cit8] Herrera R. P. and Marqués-López E., Multicomponent Reactions: Concepts and Applications for Design and Synthesis, Wiley, Hoboken, 2015

[cit9] Fares H., DiNicolantonio J. J., O'Keefe J. H., Lavie C. J. (2016). Open Heart.

[cit10] Part–IV, Studies on chalcone, http://shodhganga.inflibnet.ac.in/bitstream/10603/2162/11/11_part4.pdf, website accessed on 10th Mar 2014

[cit11] Bano T., Kumar N., Dudhe R. (2012). Org. Med. Chem..

[cit12] Kotaiah Y., Krishna N. H., Raju K. N., Rao C. V., Jonnalagadda S. B., Maddila S. (2012). J. Korean Chem. Soc..

[cit13] Elumalai K., Ali M. A., Elumalai M., Eluri K., Srinivasan S. (2013). J. Acute Disease.

[cit14] Trivedi A. R., Dodiya D. K., Ravat N. R., Shah V. H. (2008). Arkivoc.

[cit15] Khoje A. D., Kulendrn A., Charnock C., Wan B., Franzblau S., Gundersen L. L. (2010). Bioorg. Med. Chem..

[cit16] Chaudhari P. K., Pandey A., Shah V. H. (2010). Orient. J. Chem..

[cit17] Shmalenyuk E. R., Kochetkov S. N., Alexandrova L. A. (2013). Russ. Chem. Rev..

[cit18] Doan T. N., Tran D. T. (2011). Pharmacol. Pharm..

[cit19] JyothI M. V., Prasad Y. R., Venkatesh P., Sureshreddy M. (2012). Chem. Sci. Trans..

[cit20] Rao N. S., Kistareddy C., Balram B., Ram B. (2012). Pharma Chem..

[cit21] Bhalgat C. M., Irfan Ali M., Ramesh B., Ramu G. (2014). Arabian J. Chem..

[cit22] Reymond J. L., Awale M. (2012). ACS Chem. Neurosci..

[cit23] James M. J., O'Brien P., Taylor R. J. K., Unsworth W. P. (2016). Chem.–Eur. J..

[cit24] Welsch M. E., Snyder S. A., Stockwell B. R. (2010). Curr. Opin. Chem. Biol..

[cit25] Kalaria P. N., Karad S. C., Raval D. K. (2018). Eur. J. Med. Chem..

[cit26] Abbas S. E.-S., George R. F., M Samir E., Aref M. M. A., Abdel-Aziz H. A. (2019). Future Med. Chem..

[cit27] Abdel-Aziem A., El-Gendy M. S., Abdelhamid A. O. (2012). Eur. J. Chem..

[cit28] Mamaghani M., Tabatabaeian K., Araghi R., Fallah A., Hossein Nia R. (2014). Org. Chem. Insights.

[cit29] Verma A. K., Singh A. K., Islam M. M. (2014). Int. J. Pharm. Sci..

[cit30] El-Shahat M., Elhefny E. A., El-Sayed A. A., Salama M. A. M. (2015). Int. J. Pharm..

[cit31] Swami M. B., Nagargoje G. R., Mathapati S. R., Bondge A. S., Jadhav A. H., Panchgalle S. P., More V. S. (2023). J. Appl. Organomet. Chem..

[cit32] Baghayeri M., Amiri A. h., Karimabadi F., Di Masi S., Maleki B., Adibian F., Pourali A. R., Malitesta C. (2021). J. Electroanal. Chem..

[cit33] Adibian F., Pourali A. R., Maleki B., Baghayeri M., Amiri A. h. (2020). Polyhedron.

[cit34] Azizi B., Poor Heravi M. R., Hossaini Z. S., Ebadid A. G., Vessally E. (2021). RSC Adv..

[cit35] Katal R., Masudy-Panah S., Sabaghan M., Hossaini Z. S., Davood Abadi Farahani M. H. (2020). Sep. Purif. Technol..

[cit36] Ghashghaee M. , Ghambarian M. and Azizi Z., Black Phosphorus: Synthesis, Properties and Applications, 2020, pp. 59–72

[cit37] Zarei F., Soleimani-Amiri S., Azizi Z. (2022). Polycycl. Aromat. Comp..

[cit38] Ghamari Kargar P., Maleki B., Ghani M. (2024). ACS Appl. Nano Mater..

[cit39] Tayebee R., Esmaeili E., Maleki B., Khoshniat A., Chahkandi M., Mollania N. (2020). J. Mol. Liq..

[cit40] Maleki B., Nejat R., Alinezhad H., Mousavi S. M., Mahdavi B., Delavari M. (2020). Org. Prep. Proced. Int..

[cit41] Mehrizi Marvast S., Rostami E. (2024). Asian J. Green Chem..

[cit42] Zare A., Mostaghar F. (2024). Chem. Methodol..

[cit43] Soleimani-Amiri S., Hossaini Z. S., Arabkhazaeli M., Karami H., Afshari S., Abad S. (2019). J. Chin. Chem. Soc..

[cit44] Djurišić A. B., Chen X., Leung Y. H., Man A. (2012). J. Mater. Chem..

[cit45] Halliwell B. (1999). Free Radical Res..

[cit46] Babizhayev M. A., Deyev A. I., Yermakovea V. N., Brikman I. V., Bours J., Bours J. (2004). Drugs R&D.

[cit47] Liu L., Meydani M. (2002). Nutr. Rev..

[cit48] Keshvari Kenari M., Asghari S., Maleki B., Mohseni M. (2024). Res. Chem. Intermed..

[cit49] Ramezanpour-Touchahi M., Mazloumi M., Taherpour Nahzomi H., Shirini F., Tajik H. (2024). Chem. Methodol..

[cit50] Zare A., Khanivar R. (2024). Asian J. Green Chem..

[cit51] Sajjadifar S., Abakhsh F., Arzehgar Z. (2024). Chem. Methodol..

[cit52] Fathi Hasanbarogh A., Ghasemi N., Ezzatzadeh E. (2024). Int. J. Environ. Anal. Chem..

[cit53] (a) Azizi Z. , Ghashghaee M. and Ghambarian M., Black Phosphorus: Synthesis, Properties and Applications, 2020, pp. 157–169

[cit54] Ghasemi S., Badri F., Rahbar Kafshboran H. (2024). Asian J. Green Chem..

[cit55] Ghafuri H., Zargari M., Emami A. (2023). Asian J. Green Chem..

[cit56] Amini I., Azizkhani V., Ezzatzadeh E., Pal K., Rezayati S., Fekri M. H., Shirkhani P. (2020). Asian J. Green Chem..

[cit57] Moshtaghi Zonouz A., Abkar Aras M., Jafari N., Rezaei Z., Hamishehkar H. (2025). RSC Adv..

[cit58] Ebrahimzadeh P., Maleki B., Ghani M., Peiman S. (2024). Chem. Methodol..

[cit59] Malamiri F., Khaksar S., Badri R., Tahanpesar E. (2019). Curr. Org. Synth..

[cit60] Yazdan Kushkoo F., Khabazzadeh H., Khaleghi M. (2024). Chem. Methodol..

[cit61] Sohrabi-Kashani L., Zolriasatein A., Eftekhari Yekta B., Med J. (2023). Nanomater. Chem..

[cit62] Iravani N., Karami B., Asadimoghaddam F., Monfared M., Karami N. (2012). J. Sulfur Chem..

[cit63] Al-Tufah M. M., Beebaeny S., Salem Jasim S., Lateef Mohammed B. (2023). Chem. Methodol..

[cit64] Hossaini Z. S., Mohammadi M., Sheikholeslami-Farahani F. (2022). Front. Chem..

[cit65] Mahmud M., Jahangir Hossain M., Med J. (2024). Nanomater. Chem..

[cit66] Kohanfekr T., Hakimi M., Hassani H., Ali Hosseini H. (2023). Chem. Methodol..

[cit67] Alirezapour F., Bamdad K., Khanmohammadi A., Ebrahimi N. (2022). J. Mol. Model..

[cit68] Mubassir M., Parveen B. R., Singh B., Kumar A., Ahamad N., Med J. (2024). Nanomater. Chem..

[cit69] Hakimi F., Babaei S., Golrasan E. (2024). Adv. J. Chem., Sect. A.

[cit70] Soleimani-Amiri S., Koohi M., Azizi Z. (2018). J. Chin. Biochem. Soc..

[cit71] Zare A., Jazinizadeh E., Lotfifar N., Appl J. (2024). Organomet. Chem..

[cit72] Mhaibes R. M., Arzehgar Z., Heydari M. M., Fatolahi L. (2023). Asian J. Green Chem..

[cit73] Hamedani N. F., Hargalani F. Z., Rostami-Charati F. (2022). Mol. Diversity.

[cit74] Davoodi E., Tahanpesar E., Massah A. R. (2021). ChemistrySelect.

[cit75] Jalilian R., Ezzatzadeh E., Taheri A. (2021). J. Environ. Chem. Eng..

[cit76] Ezzatzadeh E., Fallah Lri Sofla S., Pourghasem E., Rustaiyan A., Zarezadeh A. (2014). J. Essent. Oil Bear. Plants.

[cit77] Rezayati S., Kalantari F., Ramazani A., Ezzatzadeh E. (2021). J. Sulfur Chem..

[cit78] Karami Hezarcheshmeh N., Godarzbod F., Faal Hamedanii N., Vaseghi S. (2023). Polycycl. Aromat. Comp..

[cit79] Farhood H. B., Radhi M. N., Hassan Z. S. (2024). J. Med. Nanomater. Chem..

[cit80] Nirwan S. A., Shinde S. A., Ughade R. A., Tadke V. B., Dhanmane S. A. (2025). Asian J. Green Chem..

[cit81] Shimada K., Fujikawa K., Yahara K., Nakamura T. (1992). J. Agric. Food Chem..

[cit82] Yen G. C., Duh P. D. (1994). J. Agric. Food Chem..

[cit83] Yildirim A., Mavi A., Kara A. A. (2001). J. Agric. Food Chem..

[cit84] Chanda A., Fokin V. V. (2009). Chem. Rev..

[cit85] Narayan S., Muldoon J., Finn M. G., Fokin V. V., Kolb H. C., Barry Sharpless K. (2005). Angew. Chem., Int. Ed..

[cit86] Butler R. N., Coyne A. G. (2016). Org. Biomol. Chem..

[cit87] Moghadam M. M., Zamani M. (2021). Comput. Theor. Chem..

[cit88] Moghadam M. M., Zamani M. (2021). Int. J. Quantum Chem..

[cit89] Sarvarian S., Zamani M. (2021). Struct. Chem..

[cit90] Saundane A. R., Nandibeoor M. K. (2015). Monatsh. Chem..

[cit91] Bidchol A. M., Wilfred A., Abhijna P., Harish R. (2011). Food Bioprocess Technol..

