# Supplementary material for: Cu/ZnO@GO promoted green synthesis of novel dipyridopyrimidines: evaluation of biological activity and theoretical study of the mechanism using a DFT method
Source: RSC Adv. 2025 Aug 26;15(36):29424–38. doi: 10.1039/d5ra04054j (PMC12379084; doi:10.1039/d5ra04054j)
Supplement: RA-015-D5RA04054J-s001 [file RA-015-D5RA04054J-s001.pdf]

This journal is © The Royal Society of Chemistry 2025

Cu/ZnO@GO promoted green synthesis of novel dipyridopyrimidines: Evaluation of biological activity and theoretical study on the mechanism using a DFT method

Elham Ezzatzadeh[a*], Nasrin Karami Hezarcheshmeh[b] and Reza Akbari

[a]*Department of Chemistry, Ard.C., Islamic Azad University, Ardabil, Iran*
[b]*Department of Chemistry, SR.C., Islamic Azad University, Tehran, Iran*
[c]*Department of Chemistry, Faculty of Basic Science, Gonbad Kavous University, Gonbad Kavous, Iran.*

Corresponding Email: Elham.Ezzatzadeh@iau.ac.ir; dr.ezzatzadeh@yahoo.com

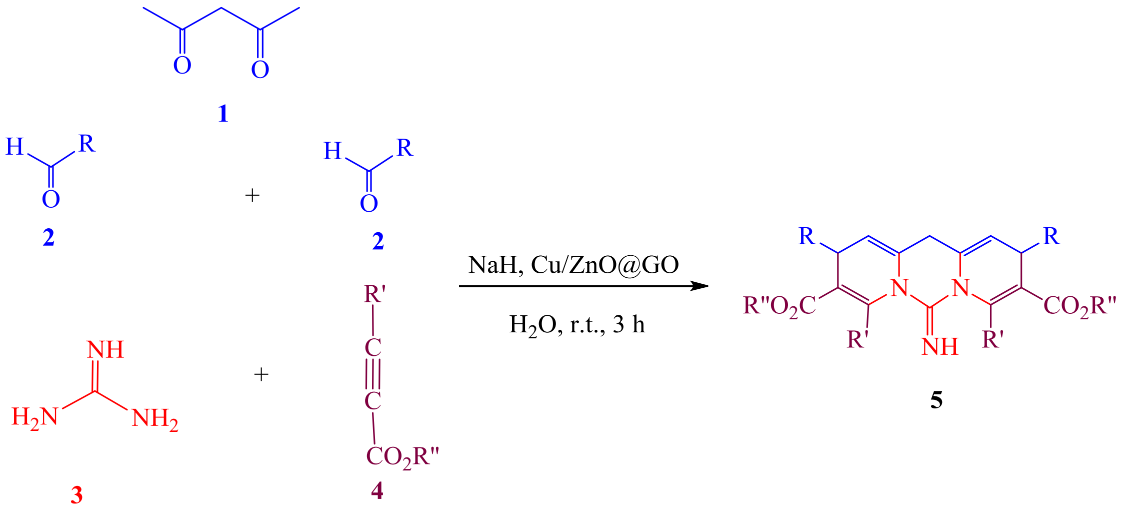

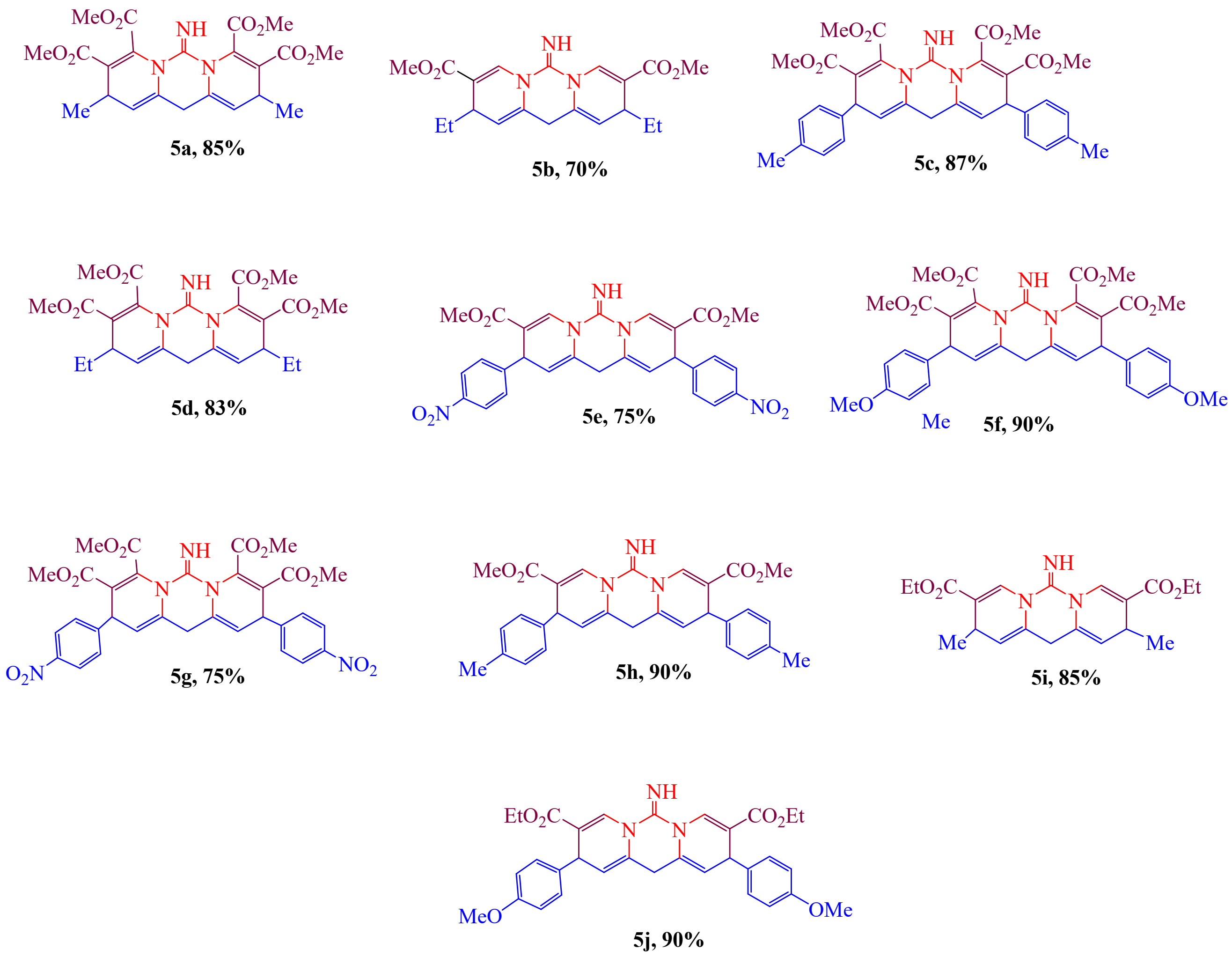

Scheme 1: Synthesis of dipyridopyrimidines **5**

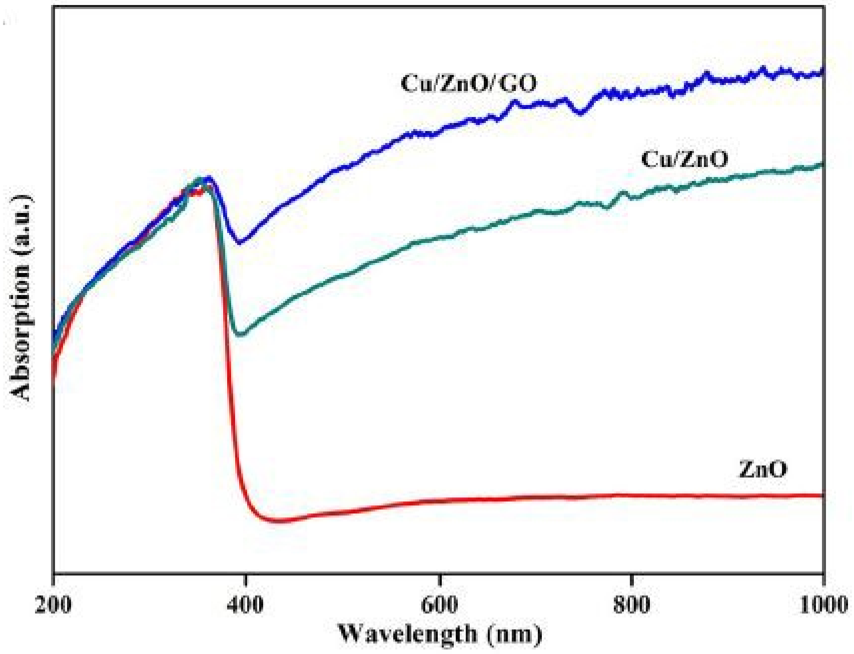

Figure 1. FT-IR (KBr) spectra of Cu/ZnO@GO

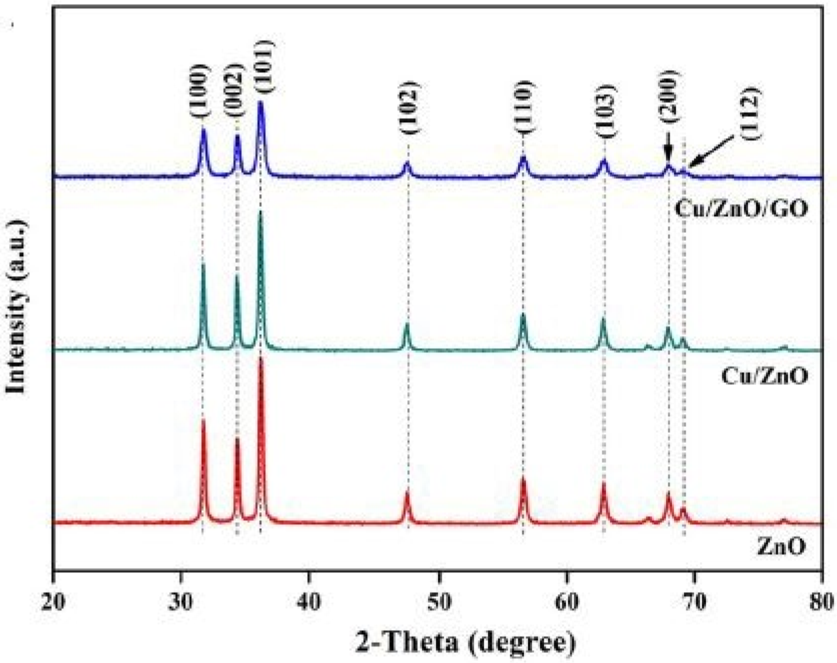

Figure 2. X-ray diffraction patterns of Cu/ZnO@GO.

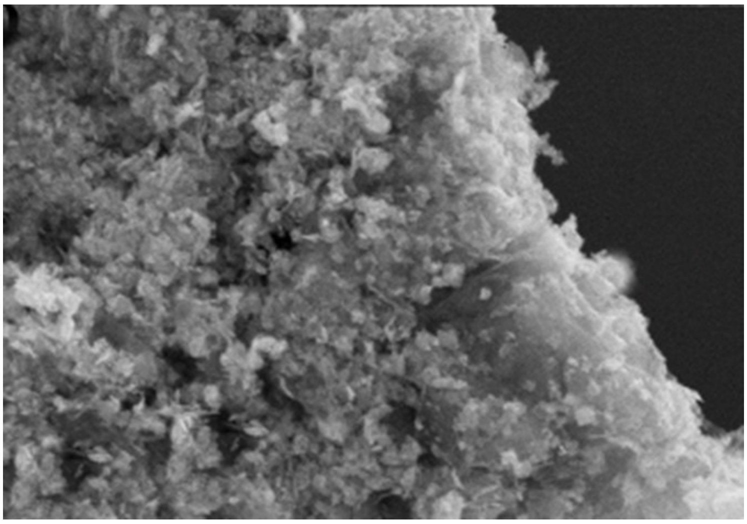

Figure 3. The FESEM analysis of Cu/ZnO@GO

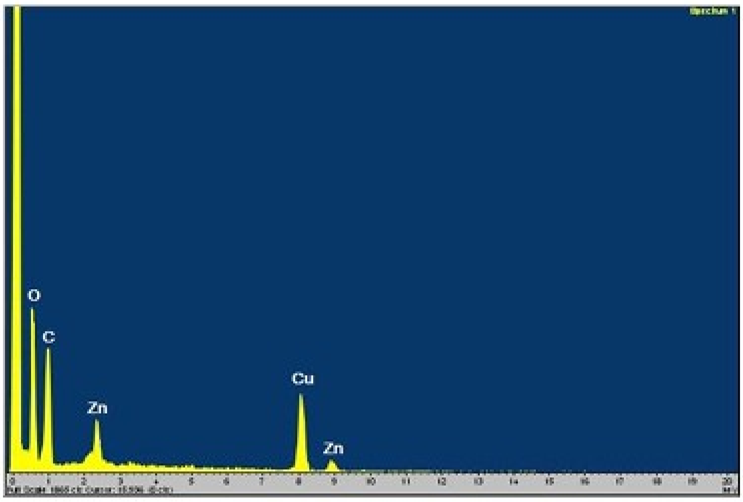

Figure 4. The EDX analysis of Cu/ZnO@GO .

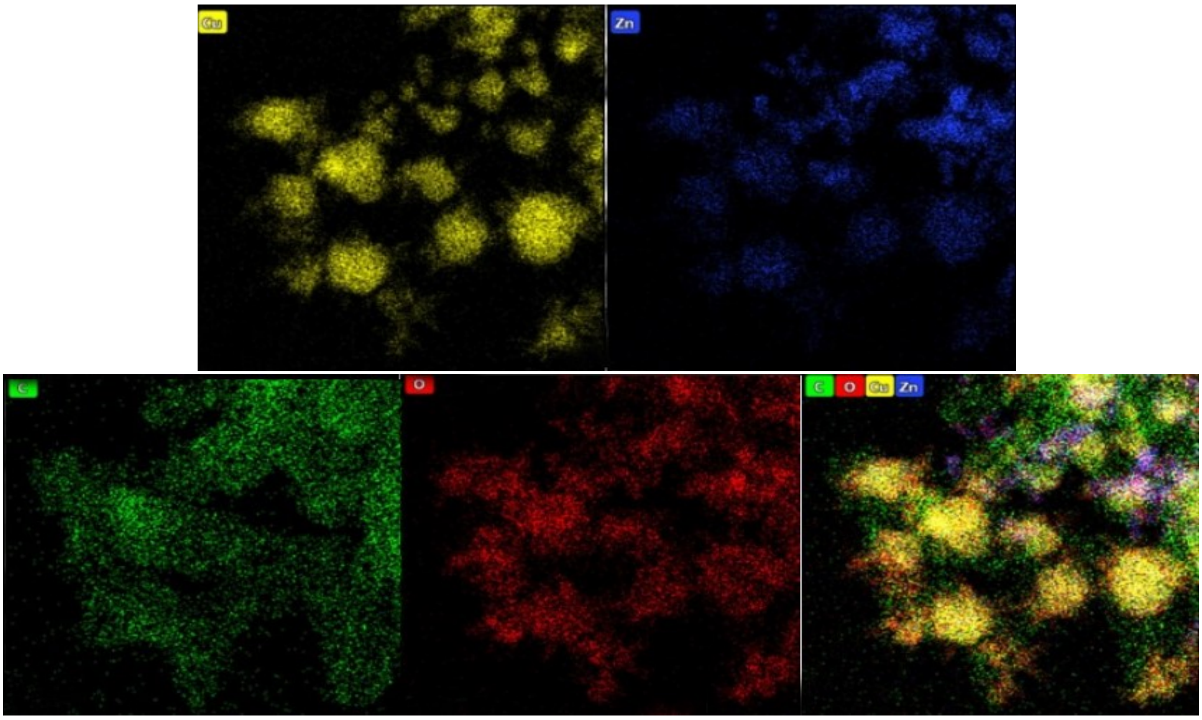

Figure 5. EDS mapping analysis of Cu/ZnO@GO

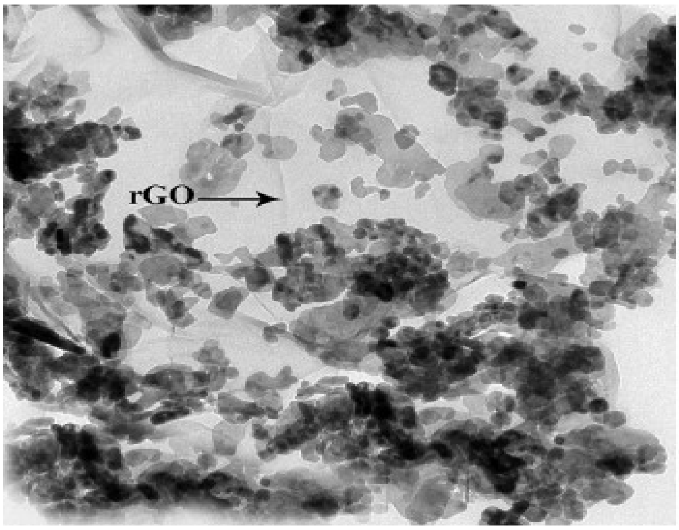

Figure 6. TEM images analysis of Cu/ZnO@GO

Table 1. Determining the best conditions, including catalyst, amount of catalyst and temperature for the synthesis of **5a**

Entry	Catalyst	Temp.(°C)	Catalyst (g)	TON	TOF	Time (h)	Yield%[a]
1	none	r.t.	-	-	-	10	trace
2	none	100	-	-	-	10	trace
3	Cu NPs	r.t.	0.01	45	15	3	45
4	ZnO NPs	r.t.	0.015	37.3	12.4	3	56
5	ZnO NPs	r.t.	0.02	32.5	10.8	3	65
6	ZnO NPs	r.t.	0.025	26	8.67	3	65
7	ZnO@MWCNTs	r.t.	0.02	35	11.67	3	70
8	MWCNTs	r.t.	0.02	12.5	4.17	3	25
9	Cu@ MWCNTs	r.t.	0.02	27.5	9.17	3	55
10	Cu/ZnO	r.t.	0.02	39	13	3	78
11	Cu/ZnO@GO	r.t.	0.02	42.5	14.17	3	85
12	Cu/ZnO@GO	100	0.02	42.5	14.18	3	85

[a] Isolated yields

Table 2. Determining the best solvent for generation of **5a**

Entry	Solvent	Time (h)	Yield%[a]
1	EtOH	15	78
2	CH_2Cl_2	8	75
3	$CHCl_3$	5	75
**4**	**H_2O**	**3**	**85**
5	Solvent-free	8	60
6	DMF	12	45
7	Toluene	12	75
8	CH_3CN	5	87

[a] Isolated yields

Table 3. Reusability of catalyst for synthesis of compound **5a**

Run	% Yield[a]
1rd	85
2rd	85
3rd	85
4rd	80
5rd	78

[a] Isolated yields

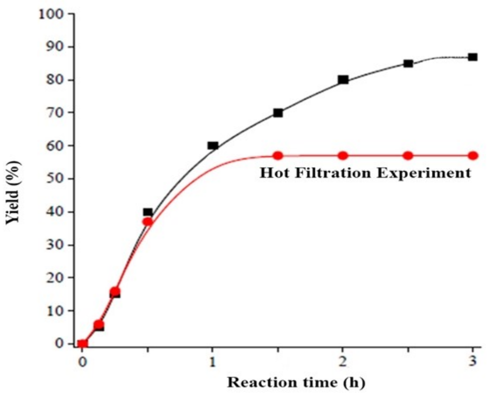

Figure 7. Hot filtration of Cu/ZnO@GO.

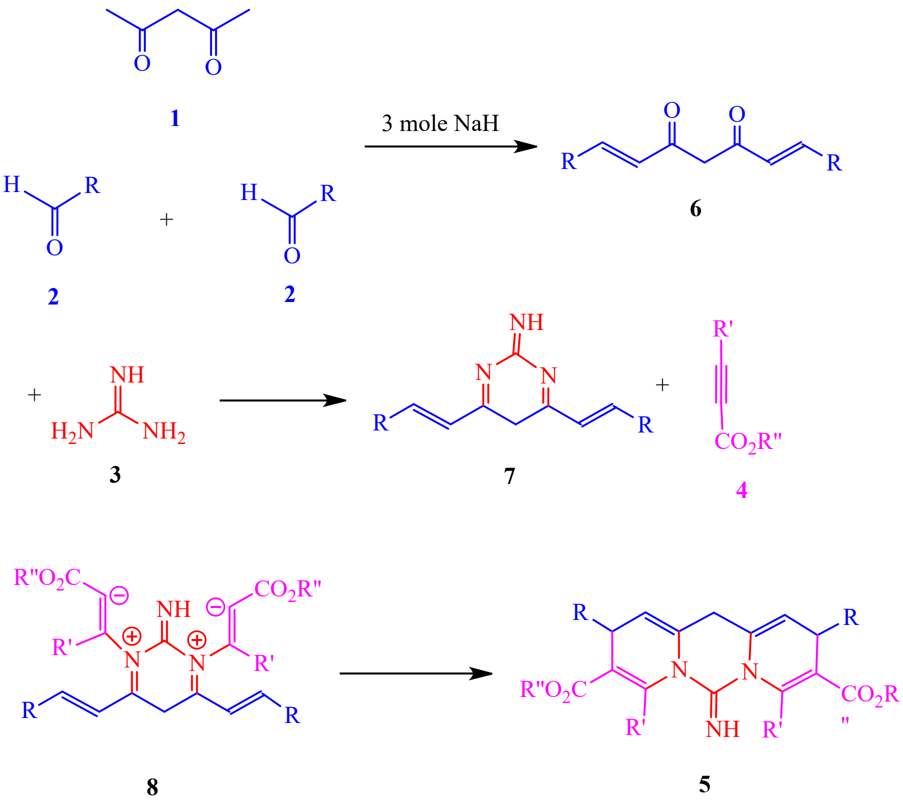

Scheme 2. Proposed mechanism for the formation of **5**

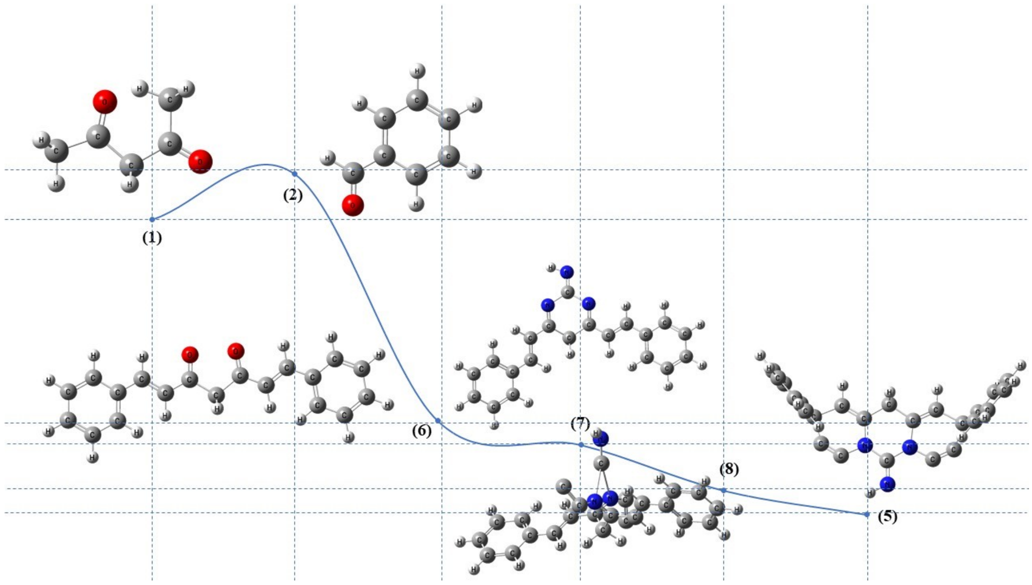

Scheme 3. Profile of the activated intermediates for the formation of **5** that calculated by B3LYP/6-311G(d,P) level of theory

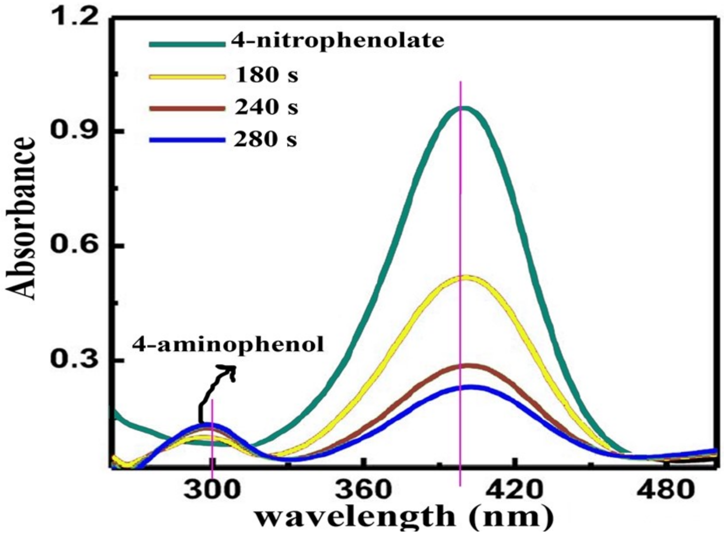

Figure 8. UV–Vis spectrum of reduction of 4-nitrophenol and photocatalytic reduction of the 4-NP

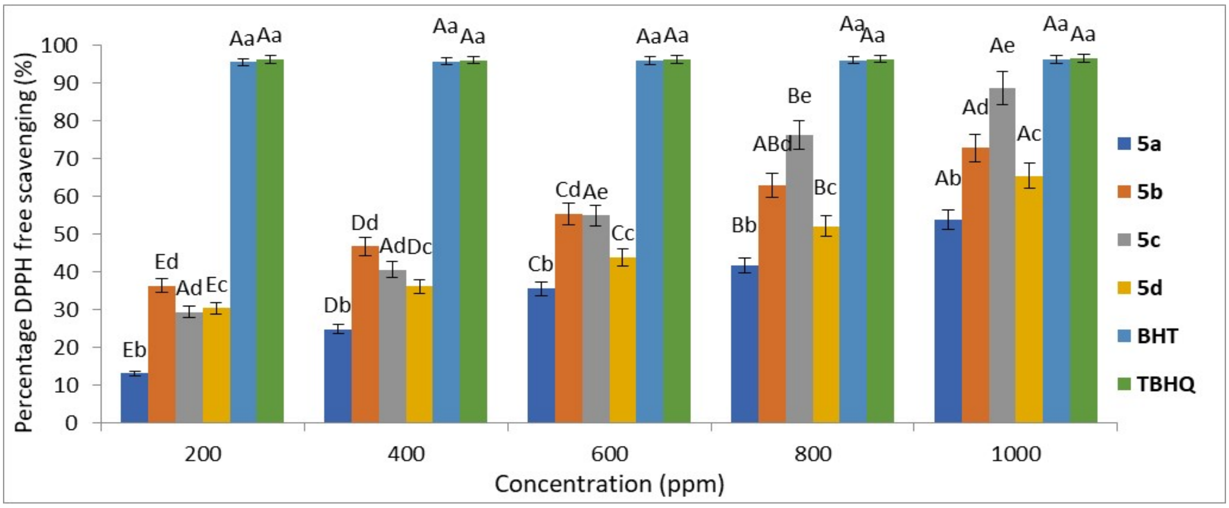

Figure 9. Order of antioxidant activity of **5a-5d** using DPPH

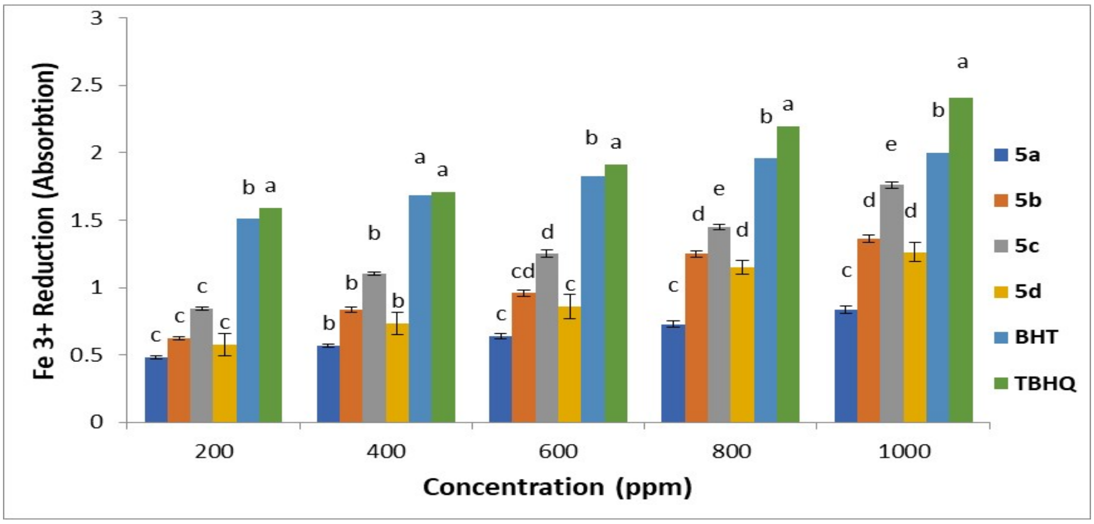

Figure 10. Ferric ions (Fe^{3+}) decreasing antioxidant ability (FRAP) of compounds **5a-5d**

Table 4. Antibacterial activity of synthesized compounds **5**

Compounds	***Staphylococcus aureus*** **(+) PTCC 1337**		***Bacillus cereus*** **(+) PTCC 1023**		***Escherichia coli*** **(-) PTCC1270**		***Klebsiella pneumoniae*** **(-) PTCC 1290**	
	**IZ(mm)[a]**	**MIC** (µg/ml)[b]	**IZ(mm)**	**MIC** (µg/ml)	**IZ(mm)**	**MIC** (µg/ml)	**IZ(mm)**	**MIC** (µg/ml)
**5a**	18±0.001	25	21±0.001	25	22±0.002	25	16±0.001	25
**5b**	10±0.002	30	8±0.002	30	10±0.003	30	7±0.001	30
**5c**	17±0.086	25	19±0.075	25	22±0.063	25	17±0.054	25
**5d**	20±0.001	20	21±0.001	20	23±0.024	20	18±0.002	20
**5e**	19±0.00	20	22±0.003	19	21±0.001	22	19±0.003	22
**5f**	10±0.001	30	9±0.001	30	9±0.00	30	8±0.012	30
**5g**	10±0.023	20	9±0.001	20	9±0.003	20	8±0.003	20
**5h**	18±0.003	25	22±0.001	25	21±0.001	25	18±0.001	25
**5i**	18±0.00	25	20±0.003	25	21±0.00	25	19±0.002	25
**5j**	12±0.001	22	10±0.00	20	10±0.014	20	9±0.003	22
**Streptomycin**	19±0.00	12.5	22±0.00	12.5	23±0.00	12.5	21±0.00	12.5
**Gentamicin**	20±0.001	12.5	24±0.00	12.5	22±0.00	12.5	20±0.001	12.5

[a]zone of inhibition in diameter in mm
[b] minimum inhibitory concentration MIC (µg/ml)
